# TMEM160 promotes tumor immune evasion and radiotherapy resistance via PD-L1 binding in colorectal cancer

**DOI:** 10.1186/s12964-024-01541-w

**Published:** 2024-03-07

**Authors:** Xiaofeng Dai, Zhipeng Wu, Ruiwen Ruan, Jingyi Chen, Chunye Huang, Wan Lei, Yangyang Yao, Li Li, Xiaomei Tang, Jianping Xiong, Miao Feng, Jun Deng

**Affiliations:** 1 Department of Oncology, The First Affiliated Hospital, Jiangxi Medical College, Nanchang University, Nanchang, Jiangxi Province 330006 China; 2 Jiangxi Key Laboratory for Individual Cancer Therapy, 17 Yongwai Street, Nanchang, Jiangxi Province 330006 China; 3 Department of Oncology, Jiangxi Provincial Chest Hospital, Nanchang, Jiangxi Province 330006 China; 4 Postdoctoral Innovation Practice Base, The First Affiliated Hospital of Nanchang University, Nanchang, 330006 People’s Republic of China

**Keywords:** TMEM160, PD-L1, SPOP, Colorectal cancer

## Abstract

**Background:**

The effectiveness of anti-programmed cell death protein 1(PD-1)/programmed cell death 1 ligand 1(PD-L1) therapy in treating certain types of cancer is associated with the level of PD-L1. However, this relationship has not been observed in colorectal cancer (CRC), and the underlying regulatory mechanism of PD-L1 in CRC remains unclear.

**Methods:**

Binding of TMEM160 to PD-L1 was determined by co-immunoprecipitation (Co-IP) and GST pull-down assay.The ubiquitination levels of PD-L1 were verified using the ubiquitination assay. Phenotypic experiments were conducted to assess the role of TMEM160 in CRC cells. Animal models were employed to investigate how TMEM160 contributes to tumor growth.The expression and clinical significance of TMEM160 and PD-L1 in CRC tissues were evaluated by immunohistochemistry(IHC).

**Results:**

In our study, we made a discovery that TMEM160 interacts with PD-L1 and plays a role in stabilizing its expression within a CRC model. Furthermore, we demonstrated that TMEM160 hinders the ubiquitination-dependent degradation of PD-L1 by competing with SPOP for binding to PD-L1 in CRC cells. Regarding functionality, the absence of TMEM160 significantly inhibited the proliferation, invasion, metastasis, clonogenicity, and radioresistance of CRC cells, while simultaneously enhancing the cytotoxic effect of CD8 + T cells on tumor cells. Conversely, the upregulation of TMEM160 substantially increased these capabilities. In severely immunodeficient mice, tumor growth derived from lentiviral vector shTMEM160 cells was lower compared with that derived from shNC control cells. Furthermore, the downregulation of TMEM160 significantly restricted tumor growth in immune-competent BALB/c mice. In clinical samples from patients with CRC, we observed a strong positive correlation between TMEM160 expression and PD-L1 expression, as well as a negative correlation with CD8A expression. Importantly, patients with high TMEM160 expression exhibited a worse prognosis compared with those with low or no TMEM160 expression.

**Conclusions:**

Our study reveals that TMEM160 inhibits the ubiquitination-dependent degradation of PD-L1 that is mediated by SPOP, thereby stabilizing PD-L1 expression to foster the malignant progress, radioresistance, and immune evasion of CRC cells. These findings suggest that TMEM160 holds potential as a target for the treatment of patients with CRC.

**Supplementary Information:**

The online version contains supplementary material available at 10.1186/s12964-024-01541-w.

## Background

Colorectal cancer (CRC) is a significant global health concern, ranking as the third most diagnosed cancer and the second leading cause of cancer-related deaths worldwide. In 2020, China reported 533,170 new CRC cases, leading to 278,476 deaths and accounting for 11.7% and 9.3% of the total cancer incidence and mortality, respectively [1]. In recent years, immune checkpoint inhibitors (ICIs) have been used to reactivate the immune system by relieving the suppressive actions that are exerted by tumors on T cells. The efficacy of ICIs has been verified and approved for clinical use in diverse advanced cancers, encompassing non-small cell lung cancer and gastric cancer [2, 3]. Moreover, ICIs have demonstrated applicability in specific patients with metastatic CRC particularly those with deficient mismatch repair or high-level microsatellite instability [4]. Nonetheless, the overall response of CRC to ICI therapy in patients with proficient mismatch repair genes or microsatellite stability has proved unsatisfactory, and the relationship between PD-L1 expression and ICI therapy in patients with CRC remains unknown [5]. Consequently, the investigation of molecular mechanisms governing PD-L1 expression in CRC is indispensable to formulate potential strategies that heighten the clinical efficacy of anti-PD-1/L1 therapy.

The transmembrane protein (TMEM) family encompasses transmembrane proteins that traverse the lipid bilayer and wield critical functions across various cell types, encompassing inflammation and store-operated Ca^2+^ entry [6, 7]. Evidence suggests that TMEM family members have close associations with tumorigenesis, invasion, and metastasis of several cancers [8–10]. TMEM160, a recently identified transmembrane protein that is localized in the mitochondrial membrane, has been implicated in neuropathic pain [11]. Furthermore, the ablation of TMEM160 triggers a mitochondrial unfolded protein response (UPR^mt^) and significantly elevates reactive oxygen species (ROS) production [12]. The relationship between TMEM160, tumorigenesis, and the tumor microenvironment is not well-understood and requires further exploration.

In this study, we report a novel association between TMEM160 and PD-L1 expression in CRC cells, which has not been previously described. Mechanistically, TMEM160 competes with SPOP for binding PD-L1 and reduces the ubiquitination-dependent degradation of PD-L1, thereby stabilizing PD-L1 expression to foster the malignant progress, radioresistance, and immune evasion of CRC cells.

## Materials and methods

### Patient samples and clinical data collection

The samples were obtained from 125 patients with CRC who underwent surgical treatment at the First Affiliated Hospital of Nanchang University from January 2018 to December 2018. New cancerous tissue and matched adjacent normal tissue samples were stored in liquid nitrogen. All patients provided written informed consent, and the research was conducted in accordance with the applicable national ethical standards. This study was approved by the Research Ethics Committee of the First Affiliated Hospital of Nanchang University (approval number (2023)CDYFYYLK(07–014)).

### Cell culture

Four human CRC cell lines, DLD1, HCT116, SW480, and RKO, were cultured in high glucose dulbecco's modified eagle medium (DMEM) containing 10% fetal bovine serum (FBS). HEK293T was cultivated in high glucose DMEM containing 10% FBS. CT26 mouse colon cancer cell were cultured in RPMI1640 medium filled with 10% FBS. All cell lines were cultivated in a moist incubator at 37℃ and 5% CO_2_.

### RNA interference, lentivirus, plasmid construction, and transfection

To knockdown TMEM160, siRNA was utilized. The detailed sequence targeting TMEM160 and lentivirus vector is shown below: CAUGCAGAGUGACAUGGGUTTACCCAUGUCACUCUGCAUGTT;CGAGGACUGGGACAUUAAATTUUUAAUGUCCCAGUCCUCGTT. For siRNA transfection, the cells were grown to a concentration of 50–60% and subsequently transfected with the respective siRNA constructs using TurboFect Transfection Reagent (R0531, Thermo Fisher Scientific Corp, Waltham, MA, USA) according to the manufacturer's instructions. For gene transfection, the cells were grown to a concentration of 70–80% and transfected with plasmids containing TMEM160 cDNA using TurboFect Transfection Reagent. Cell proteins or total RNA were extracted 48 h after transfection to assess the efficiency of knockdown or overexpression.

### RNA extraction and RT-qPCR analysis

RNA extraction reagents (R701-01, Vazyme Corp, Nanjing, China) were used to extract RNA from the cell samples when the cells were grown to a concentration of 85%–95%.Synthesis of SuperMix by transcriptional All-in-One first strand cDNA Transgen(AT341-01, TransGen Biotech Corp, Beijing, China.) was used to reverse transcribe the dissolved RNA into complementary DNA (cDNA). RT-qPCR was performed using a StepOnePlus Real-Time PCR System (11736059, Thermo Fisher Scientific Corp, Waltham, MA, USA).GAPDH was used for normalization, and the test was repeated three times for each sample. The primer sequences for GAPDH, TMEM160, and PD-L1 were as follows:

- GAPDH-S: 5′-GGAAGCTTGTCATCAATGGAAATC-3′
- GAPDH-A: 5′-TGATGACCCTTTTGGCTCCC-3′
- TMEM160-S: 5′-TCCTCTCCTGGTTCCGCAA-3′
- TMEM160-A: 5′-CCCAGCAGGAAGAAGCCATAT-3′
- PDL1-S: 5′-GCCGAAGTCATCTGGACAAGC-3′
- PDL1-A: 5′-GTGTTGATTCTCAGTGTGCTGGTCA-3′

### Western blotting

For protein immunoblotting analysis, we extracted total protein from the cells 48 h after transfection, and the detailed procedure followed that of our previous work [13, 14]. Western blotting was performed using the following primary antibodies: TMEM160 (ab185451, Abcam Corp, Cambridge, UK), PD-L1 (66248–1-Ig, Proteintech Group Corp, Rosemont, USA), SPOP (16750–1-AP, Proteintech Group Corp, Rosemont, USA), FLAG (66008–4-Ig, Proteintech Group Corp, Rosemont, USA), HA (51064–2-AP, Proteintech Group Corp, Rosemont, USA), Myc (16286–1-AP, Proteintech Group Corp, Rosemont, USA), His (66005–1-Ig, Proteintech Group Corp, Rosemont, USA), CD8A (TA374492, ORIGENE Corp, Wuxi, China), and GAPDH (60004–1-Ig, Proteintech Group Corp, Rosemont, USA). The antibodies were used according to the manufacturers’ protocols.

#### Molecular docking

To assess the potential direct interaction between TMEM160 and PD-L1 proteins based on their three-dimensional (3D) spatial structures, we acquired the 3D structure of PD-L1 from the Protein Data Bank (PDB) and predicted the 3D structure model of TMEM160 using data from the UniProt database. Molecular docking was performed using the Discovery Studio software, developed by BIOVIA Company in the United States. The protein was pre-processed using Discovery Studio software, and the original ligands was extracted from their structures. Subsequently, a molecular docking analysis between TMEM160 and PD-L1 was conducted using the ZDOCK module. The binding conformation of the protein–protein complexes was optimized using the RDOCK module. Finally, the Analysis tool was utilized to visualize the protein–protein complexes. This analysis allowed us to evaluate the possible binding and spatial arrangement of TMEM160 and PD-L1, providing insight into their latent interaction.

### Co-immunoprecipitation assay

By conducting co-immunoprecipitation (Co-IP) assays on DLD1 and HCT116 cells, we analyzed the binding relationship between TMEM160, SPOP, and PD-L1. We used the Protein A/G Immunoprecision Kit (22202–100, Beaver bio Corp, Suzhou, China) according to the protocol of the manufacturer for Co-IP. As in our previous work [15], we performed a western blotting analysis on the proteins that were removed from the beads.

### Ubiquitination assay

To detect the ubiquitination of PD-L1 protein, Flag-PD-L1, Myc-TMEM160, HA-SPOP, and His-Ub plasmids were transfected into HCT116 cells, which were treated with MG132 (10 μM) for 6 h before lysis. The cell lysate was mixed with Flag beads and incubated at 4℃ for one night. Wash Buffer was then used to wash the beads three times, and 1X Loading Buffer was added to boil the beads and collect the immunoprecipitated proteins. The experimental program is detailed in previous reports [16, 17].

### GST pull-down assay

To determine whether TMEM160 directly binds to PD-L1, we conducted GST pull-down experiments using a GST Pull-Down Kit (M7006, Mabnus Corp, Wuhan, China) according to the manufacturer's protocol. The purified proteins used were Recombinant Human PD-L1 (C315, Novoprotein Corp, Suzhou, China), TMEM160 Fusion Protein (Ag24915, Proteintech Group Corp, Rosemont, USA), and GST Tag Fusion Protein (Ag0040, Proteintech Group Corp, Rosemont, USA). The extracted protein was analyzed by gel electrophoresis and western blot.

### Flow cytometry analysis

The cells were initially rinsed with Phosphate Buffer Saline (PBS) and subsequently stained for cell surface PD-L1. This staining process was conducted on ice for 30 min using a solution of PBS supplemented with 20% FITC (Fluorescein Isothiocyanate) labeled antibody (558065, Univ Crop, Shanghai, China). After the staining period, the cells were washed again with PBS to remove any unbound antibodies. Subsequently, the samples were analyzed using a Mindray flow cytometer (BriCyte E6, Mindray Corp, Nanshan District, Shenzhen, China), and the data analysis was performed using FlowJo software (FlowJo ™10 software, Becton, Dickinson and Company, Franklin Lake, New Jersey, United States).

#### CHX half-life assay

To explore the influence of TMEM160 expression on the half-life of PD-L1, we conducted two separate experiments. Firstly, we knocked out TMEM160 in DLD1 cells, and secondly, we overexpressed TMEM160 in SW480 cells. Subsequently, we treated the cells with CHX (HY-12320, MedChemExpress Corp, New Jersey, USA) at a final concentration of 40 μg/ml. The cells were treated for different durations (0, 2, 4, and 8 h) before protein extraction. Western blot analysis was performed to assess the impact of TMEM160 on the half-life of PD-L1.

#### MG132 rescue assay

To investigate the underlying mechanism through which TMEM160 stabilizes PD-L1 protein expression, we performed a knockdown of TMEM160 in HCT116 cells. Subsequently, we treated the cells with MG132 (HY-13259, MedChemExpress Corp, New Jersey, USA) at a final concentration of 10 μM. After a 6-h treatment, protein extraction was carried out, followed by Western blot analysis. Our objective was to determine whether MG132 could restore the reduced PD-L1 expression resulting from TMEM160 deficiency.

### Cell proliferation and colony formation

Cell proliferation was evaluated using Cell Count Kit 8 (CCK-8) (GK10001, GlpBio Corp, Montclair, CA, USA). The CCK-8 assay measures cell viability by analyzing the absorbance of samples at 450 nm. To assess the effect of TMEM160 expression on CRC cell colony formation, cloning experiments were conducted following established protocols [18, 19]. To ensure the accuracy and reliability of the results, we repeated each experiment three times.

### Migration and invasion assays

For migration and invasion assays, transfected cells (2 × 10^5^) were seeded in the upper chamber of transwell inserts and cultured with 200 μL of serum-free medium. A complete culture medium (basic medium + 10% FBS) was added to the lower chamber. After 24 h of cultivation, the medium was replaced with fresh medium. After 48 h of cultivation, the medium was removed and cleaned three times with PBS. The cells were then fixed with 1 mL of 4% paraformaldehyde (PFA) in each well, the PFA was removed after 30 min, 1 mL of crystal violet staining solution was added, and the solution was incubated for another 30 min. The bottom of the chamber was washed three times with water, air-dried, and photographed. Notably, the transwell inserts used in the invasion assay were pre-coated with a matrix gel and mixed with a pre-chilled basic medium at a 1:8 ratio before cell seeding. We conducted each experiment three times.

### Immunofluorescence

Immunofluorescence(IF) assays were performed to evaluate the effect of TMEM160 on PD-L1 expression in CRC cells. The cells were cultured on glass slides for 24 h, then washed twice with PBS, fixed in 4% PFA for 15 min, and washed twice with PBS, each time for 5 min. The cells were placed in 2% Triton for 10 min, then rinsed with PBS twice for 5 min each time. Blocking was performed with 10% bovine serum albumin (BSA) for 30 min and the cells were then washed twice with PBS for 5 min each. The cells were then incubated with the corresponding primary antibody at 4℃ overnight. The cells were washed with PBS three times, for 5 min each time, and then incubated with the corresponding secondary antibodies in the dark room for 30 min. Subsequently, the cells underwent nuclear staining and were incubated with 4-amino-6-diamino-2-phenylindole (DAPI) and an anti-fluorescent quenching agent was added. Finally, the cellular localization of PD-L1 and TMEM160 was examined using confocal microscopy.

### T cell-mediated tumor cell killing

DLD1 cells that were stably expressing either shScr (control) or shTMEM160#1 were seeded in 24-well plates at a density of 10^5^ cells per well. Human Jurkat cells were activated with 25 ng/mL of PMA (Phorbol 12-myristate 13-acetate) and 1 μg/mL of PHA (Phytohemagglutinin) obtained from CSNpharm for a duration of 48 h. Following activation, the activated Jurkat cells were added to the culture medium of DLD1 cells at a ratio of 2:1 (Jurkat cells: DLD1 cells). After incubating these cells together for 48 h, tumor cells were collected and stained with Annexin V and propidium iodide (A211-01, Vazyme Corp, Nanjing, China). After staining, Mindray flow cytometer was used to analyze the cell apoptosis rate.

### Homologous tumor xenograft mouse model

This study complied with the ethical principles of experimental animal welfare and the protocol for the animal experiments was approved by the animal welfare committee of the First Affiliated Hospital of Nanchang University (approval No: CDYFY-IACUC-202211QR022). Female nude mice (aged 4–6 weeks) were used as the experimental subjects. HCT116 colon cancer cells expressing LV-shTMEM160#1 and LV-shScr plasmid stable clones were injected subcutaneously into the right axilla of nude mice (12 mice were randomly divided into two groups, with 1 × 10^6^ cells per mouse in each group).

Female BALB/c mice were used as the experimental subjects. CT26 mouse colon cancer cells expressing LV-shTMEM160#1 and LV-shScr plasmid stable clones were injected subcutaneously into the right axilla of the BALB/c mice (12 mice were randomly divided into two groups, with 1 × 10^6^ cells per mouse in each group).

Tumor size was measured with calipers every 3 days and tumor volume was calculated using the formula: V = (L × W^2^)/2, where V, L, and W represent the tumor volume, longest diameter,and shortest diameter, respectively. The mice were sacrificed using cervical dislocation on the 22nd day. The expression of TMEM160 and PD-L1 in tumor and paracancerous tissues was detected by western blotting,and immunohistochemistry(IHC) was used to assess the expression of TMEM160 and PD-L1 and the infiltration of CD8 + cytotoxic T cells in tumor tissue and adjacent normal tissue.

### Immunohistochemistry and histopathological analysis

CRC tissue blocks were obtained from 125 CRC tumor specimens from the First Affiliated Hospital of Nanchang University in 2018. For the use of these tumor wax specimens, our study obtained approval from the Research Ethics Committee of the First Affiliated Hospital of Nanchang University (approval number (2023)CDYFYYLK(07–014)). IHC analysis was conducted following the previously described method [20]. A total of 125 clinical CRC tissue sections were subjected to IHC analysis. The immunostaining index was evaluated and independently scored by two pathologists, taking into account the staining intensity and the proportion of tumor cells showing positive staining [18].

The immunohistochemical grading of TMEM160 was determined based on staining intensity and the density of positive cells. The grading system assigned the following categories: "-", indicating negative staining; " + ", representing weak staining; " +  + ", indicating intermediate staining; and " +  +  + ", representing strong staining. The density of positive cells was assessed as follows: 0% = 0, 1–25% = 1, 26–50% = 2, 51–75% = 3, and > 76% = 4. To calculate the immunoreactivity score, the H-score, the following formula was applied: H-score = (% of staining positive cells) (0–4) × (staining intensity) (0–3). An H-score ranging from 0 to 4 was considered TMEM160 low, while an H-score ranging from 5 to 12 was considered TMEM160 high. PD-L1 expression was categorized using the TPS (Tumor Proportion Score), which represents the percentage of tumor cells displaying membranous PD-L1 staining [21]. The quantification of intraepithelial CD8 + tumor-infiltrating T lymphocytes (TILs) was performed as described by Hamanishi et al. [22]. Three independent areas with the highest TILs infiltration were selected under a microscope at 200 × magnification. The number of intraepithelial CD8 + TILs was manually counted and expressed as cells per mm^2^.

### Statistical analysis

At least three independent experiments were used for data analysis. For comparisons between two groups, two-tailed t-tests were employed. To compare two or more groups, we used a two-way analysis of variance (ANOVA). In evaluating clinical data, we used the Student's t-test when comparing continuous variables between the two groups. When assessing categorical variables, we used the chi-square (χ 2) test. Using SPSS software for statistical analysis (version 19.0; IBM Corp, Armonk, New York, USA). A *p*-value of < 0.05 was considered statistically significant.

## Results

### TMEM160 interacts with PD-L1

**Fig. 1:**
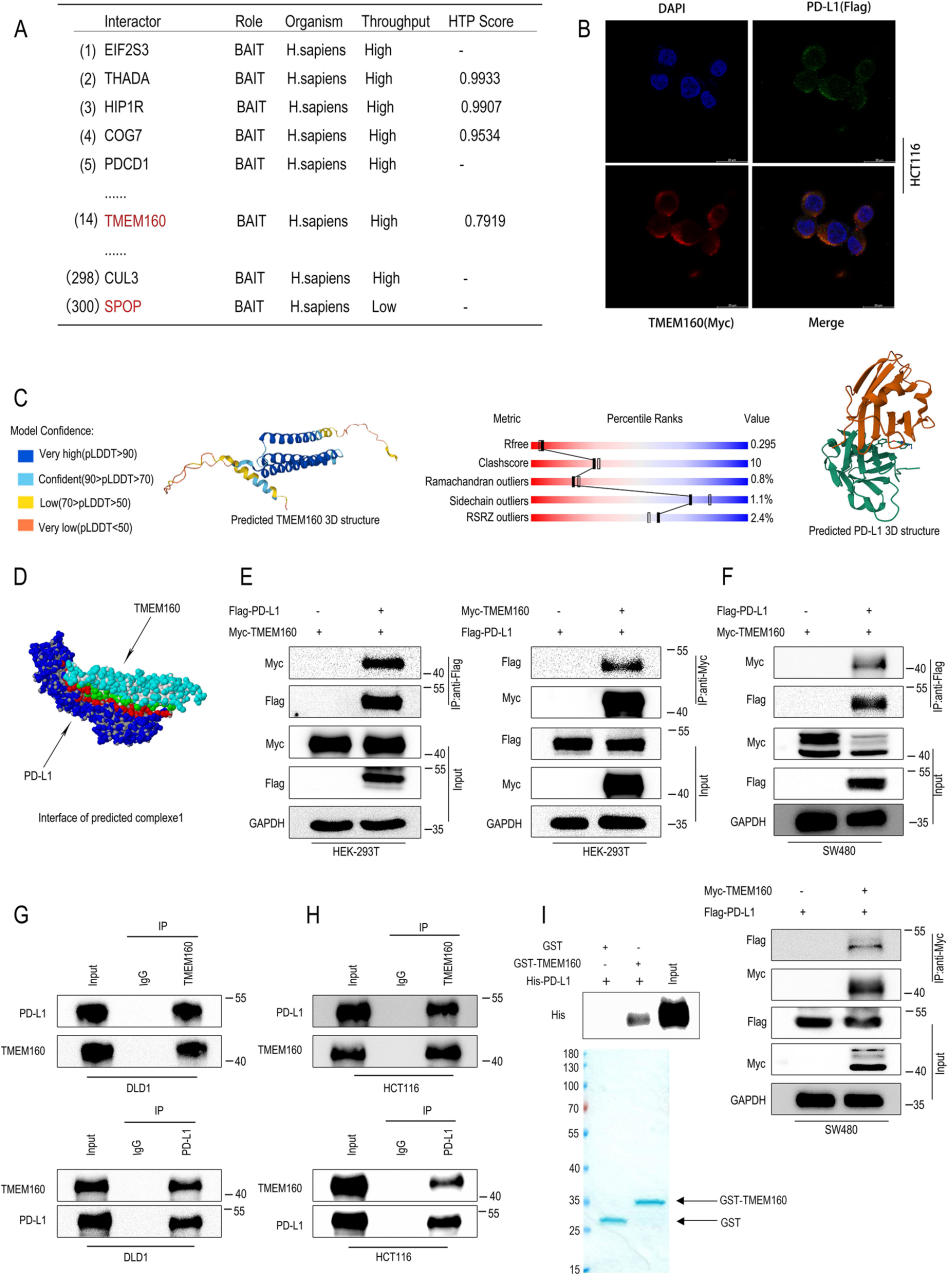
TMEM160 interacts with PD-L1. **A** The BioGRID database was used to search for the interacting proteins of CD274. **B** HCT116 cells were transfected using Flag-PD-L1 and Myc-TMEM160 for immunostaining with Flag antibodies (green), Myc antibodies (red), and DAPI antibodies (blue). Scale bar, 20 µm. **C** A prediction model for TMEM160 was obtained from the UniProt database (left), while a prediction model for PD-L1 was obtained from the Protein Data Bank (PDB) database (right). **D** The predicted interface of one of the complexes formed by TMEM160 and PD-L1. **E**, **F** Results of Co-IP of exogenous PD-L1 and TMEM160 in HEK-293 T and SW480 cells. **G**, **H** Endogenous Co-IP assays were performed in DLD1 and HCT116 cells to assess the interaction of PD-L1 with TMEM160. **I** The GST pull-down assay was conducted to confirm the direct binding between the His-PD-L1 and the GST-TMEM160 fusion protein

To evaluate the PD-L1 binding proteins, we first predicted the interacting proteins of PD-L1 in the protein interaction database (BioGRID), and the results showed that TMEM160 may interact with PD-L1 (Fig. 1A). Subsequently, a series of experiments were conducted to confirm the interaction between these two proteins. First, we performed IF experiments and found that Flag-PD-L1 co-localized with Myc-TMEM160 in the plasma membrane and cytoplasm of cancer cells (Fig. 1B). Subsequently, the 3D structure of PD-L1 was obtained from the Protein Data Bank (PDB), while the 3D structure model of TMEM160 was predicted using the UniProt database (Fig. 1C). The predicted TMEM160 model displayed satisfactory quality, considering parameters such as quality assessment, template matching, and amino acid residue characteristics. To explore their potential interaction, molecular docking was conducted using the Discovery Studio software. The results revealed a complex formation between TMEM160 and PD-L1, which was supported by their compatible protein spatial structures as depicted in Fig. 1D.

To validate the interaction between TMEM160 and PD-L1, we carried out the Co-IP experiment. Exogenous Myc-TMEM160 and Flag-PD-L1 were shown to interact in HEK-293 T and SW480 cells (Fig. 1E, F). Endogenous TMEM160 and PD-L1 also interacted with each other in DLD1 and HCT116 cells (Fig. 1G, H). GST pull-down experiments indicated that His-PD-L1 can bind to GST-TMEM160, demonstrating that TMEM160 directly interacts with PD-L1 in CRC cells (Fig. 1I).These results indicate that TMEM160 is an interacting protein of PD-L1.

### TMEM160 stabilizes PD-L1 protein expression by inhibition of ubiquitination-dependent degradation of PD-L1

**Fig. 2:**
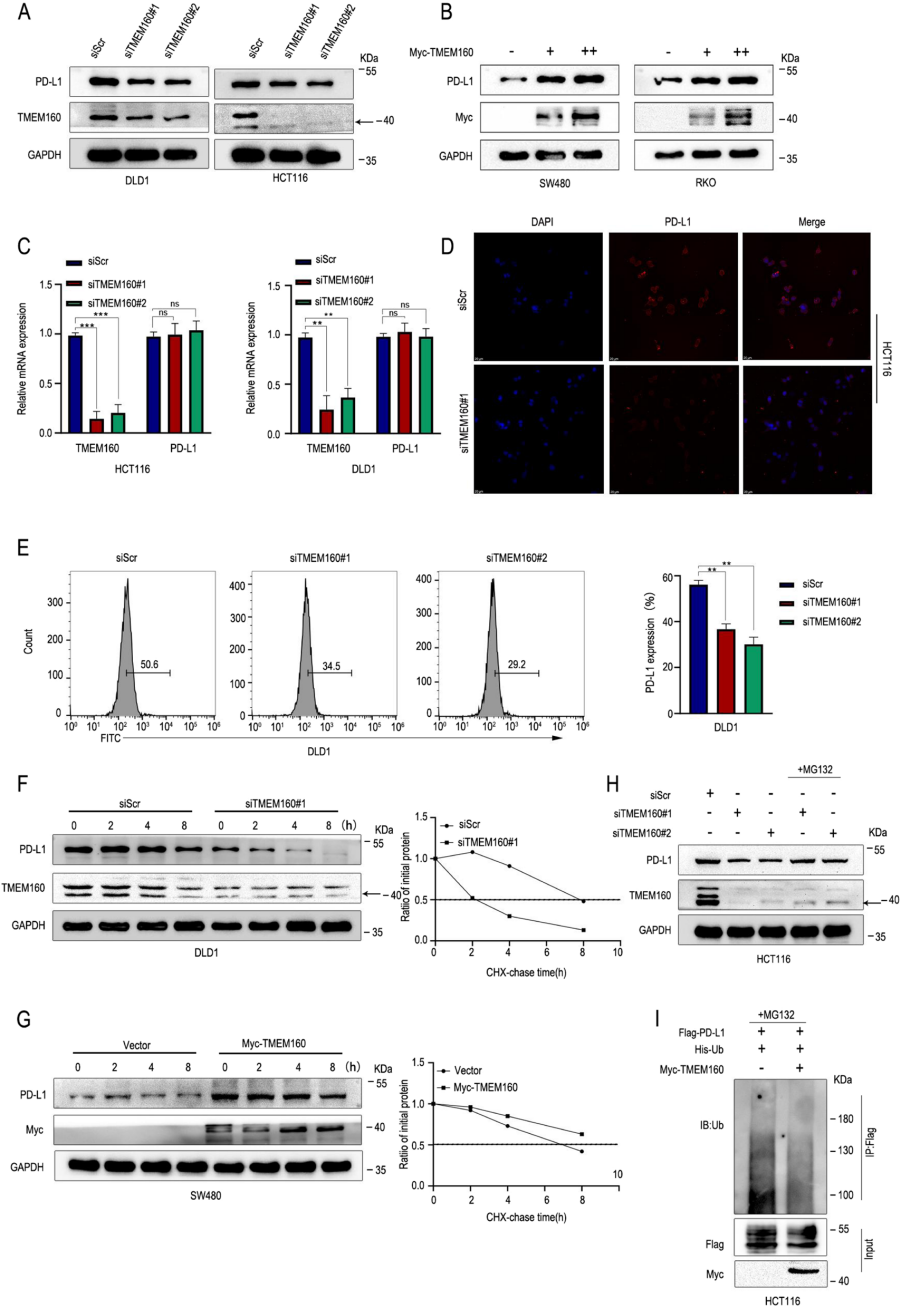
TMEM160 stabilizes PD-L1 protein expression by inhibition of ubiquitination-dependent degradation of PD-L1. **A**, **B** Western blotting assays and **C** RT-qPCR were performed to test the expression of TMEM160 and PD-L1 at the protein and mRNA levels, respectively. The expression analysis was performed following interference with TMEM160 expression using two different siRNAs (#1 and #2) in HCT116 and DLD1 cells, as well as TMEM160 overexpression in SW480 and RKO cells. **D** IF experiments of HCT116 cells transfected with siScr or siTMEM160#1 plasmid were assessed with PD-L1 expression by the fluorescence intensity of PD-L1. **E** Flow cytometric analysis of DLD1 cells transfected with siScr or siTMEM160#1 plasmid were assessed with surface PD-L1 expression. **F**, **G** To assess the effect of TMEM160 expression on PD-L1 stability, the CHX assay was performed in DLD1 cells transfected with siTMEM160#1 and SW480 cells with Myc-TMEM160. **H** Western blotting assay detected that MG132 can reverse the reduced expression of PD-L1 due to TMEM160 downregulation. **I** The ubiquitination assay was conducted in HCT116 cells to analyze the impact of TMEM160 overexpression on PD-L1 ubiquitination

To further verify the regulatory relationship between TMEM160 and PD-L1, we used siRNA to knockdown TMEM160 expression in DLD1 and HCT116 cells and observed a decrease in PD-L1 expression using western blotting (Fig. 2A). In contrast, overexpression of TMEM160 significantly increased the expression of PD-L1 in SW480 and RKO cells (Fig. 2B). Moreover, qRT-PCR showed that TMEM160 promoted the protein expression of PD-L1 without affecting its mRNA expression, indicating that TMEM160 regulates the expression of PD-L1 at the post-transcriptional level (Fig. 2C). The IF results showed that TMEM160 knockdown significantly reduced the fluorescence intensity of PD-L1 localized to the plasma membrane of HCT116 cells (Fig. 2D). Flow cytometry analysis also revealed a significant decrease in the expression of surface PD-L1 in DLD1 cells after TMEM160 knockdown (Fig. 2E).

Research has demonstrated that post-translational modifications (PTMs), such as ubiquitination, phosphorylation, glycosylation, and palmitoylation, play crucial roles in the regulation of PD-L1 [23–25]. Subsequently, we conducted an analysis of the half-life of PD-L1 to investigate the influence of TMEM160 on the stability of PD-L1 protein expression. The results revealed significant findings. In cells where TMEM160 was knocked down (DLD1 cells), the half-life of PD-L1 was notably shortened (Fig. 2F). Conversely, in cells with TMEM160 overexpression (SW480 cells), the degradation of PD-L1 was slowed down, as shown in Fig. 2G. To gain further insights into the underlying mechanism, western blotting experiments were carried out. Specifically, we treated CRC cells (HCT116) with the proteasome inhibitor MG132. The results demonstrated that the reduced expression of PD-L1 triggered by TMEM160 knockdown was restored upon MG132 treatment (Fig. 2H). These findings indicate that TMEM160 is involved in regulating the stability of PD-L1 protein expression, possibly by participating in the proteasomal degradation pathway. Further ubiquitination results showed that TMEM160 significantly reduced the ubiquitination-dependent degradation of PD-L1 (Fig. 2I), indicating that TMEM160 stabilizes PD-L1 expression through the ubiquitin proteasome pathway.

### TMEM160 competitively binds to PD-L1 with SPOP, inhibiting its ubiquitination-dependent degradation

**Fig. 3:**
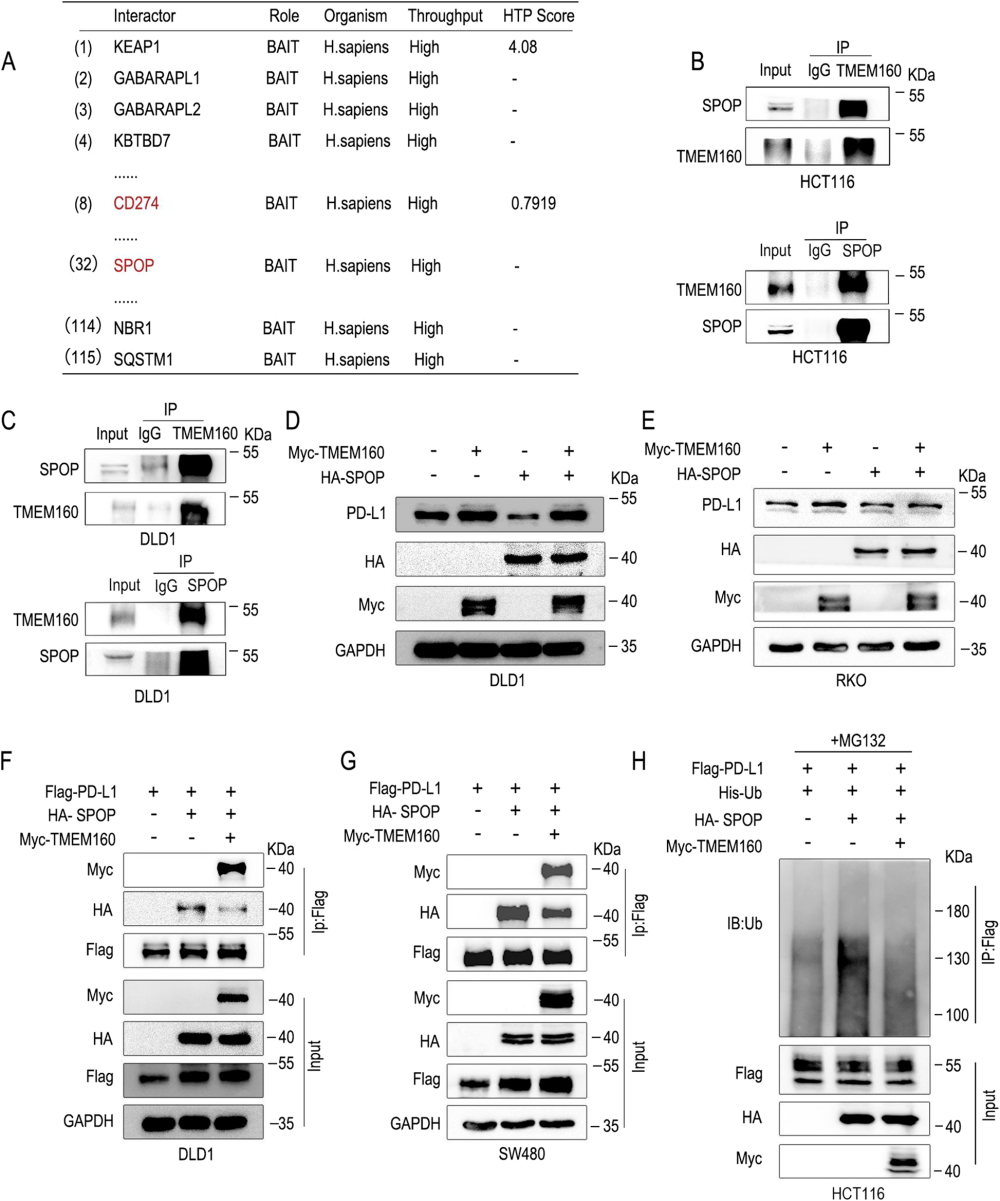
TMEM160 competitively binds to PD-L1 with SPOP, inhibiting its ubiquitination degradation. **A** The BioGRID database was used to search for the interacting proteins of TMEM160. **B**, **C** Endogenous Co-IP assays were performed in DLD1 and HCT116 cells to assess the interaction of TMEM160 with SPOP. **D**, **E** DLD1 and RKO cells were collected for western blotting analysis to investigate the effects of SPOP and TMEM160 overexpression on the expression PD-L1 in these cells. The whole-cell lysates were collected 48 h after transfection. **F**, **G** The Co-IP assay demonstrated that TMEM160 competes with SPOP for binding to PD-L1 in DLD1 and SW480 cells. **H** The results of the ubiquitination assay showed that competitive binding of TMEM160 to PD-L1 with SPOP reduces the ubiquitination degradation of PD-L1

SPOP is a key component of cullin-RING E3 ligases, known for its role in recognizing and targeting specific substrates for degradation. SPOP has been extensively studied and has shown a dual role in tumorigenesis and cancer progression [26, 27]. Numerous studies have identified SPOP as an inhibitor of various human malignancies, including lung, colon, and gastric cancers [28–30]. Using the protein interaction database, we found that TMEM160 could also interact with SPOP (Fig. 3A), which is an E3 ubiquitination-degrading enzyme of PD-L1 [31]. First, we conducted Co-IP experiments to evaluate the interaction between TMEM160 and SPOP and found that TMEM160 interacted with SPOP in HCT116 and DLD1 cells (Fig. 3B, C). Western blotting revealed that TMEM160 counteracts the inhibitory effect of SPOP on PD-L1 (Fig. 3D, E). Moreover, given that TMEM160 and SPOP interact with PD-L1, we hypothesized that TMEM160 and SPOP might competitively interact with PD-L1. The interaction between SPOP and PD-L1 was markedly reduced when TMEM160 was overexpressed in DLD1 and SW480 cells (Fig. 3F, G). Subsequently, we extended the ubiquitination experiment and found that TMEM160 antagonized the ubiquitination-dependent degradation of PD-L1 by SPOP, resulting in reduced PD-L1 ubiquitination (Fig. 3H). These results suggest that TMEM160 competitively binds PD-L1 with SPOP, thereby preventing SPOP-mediated ubiquitination-dependent degradation of PD-L1.

### TMEM160 promotes malignant biological behavior in CRC cells

To gain insights into the biological function of TMEM160, we conducted in vitro experiments using CRC cells in which TMEM160 expression was either downregulated or overexpressed. Phenotypic analyses were performed to evaluate the effects of TMEM160 on cellular behaviors.

**Fig. 4:**
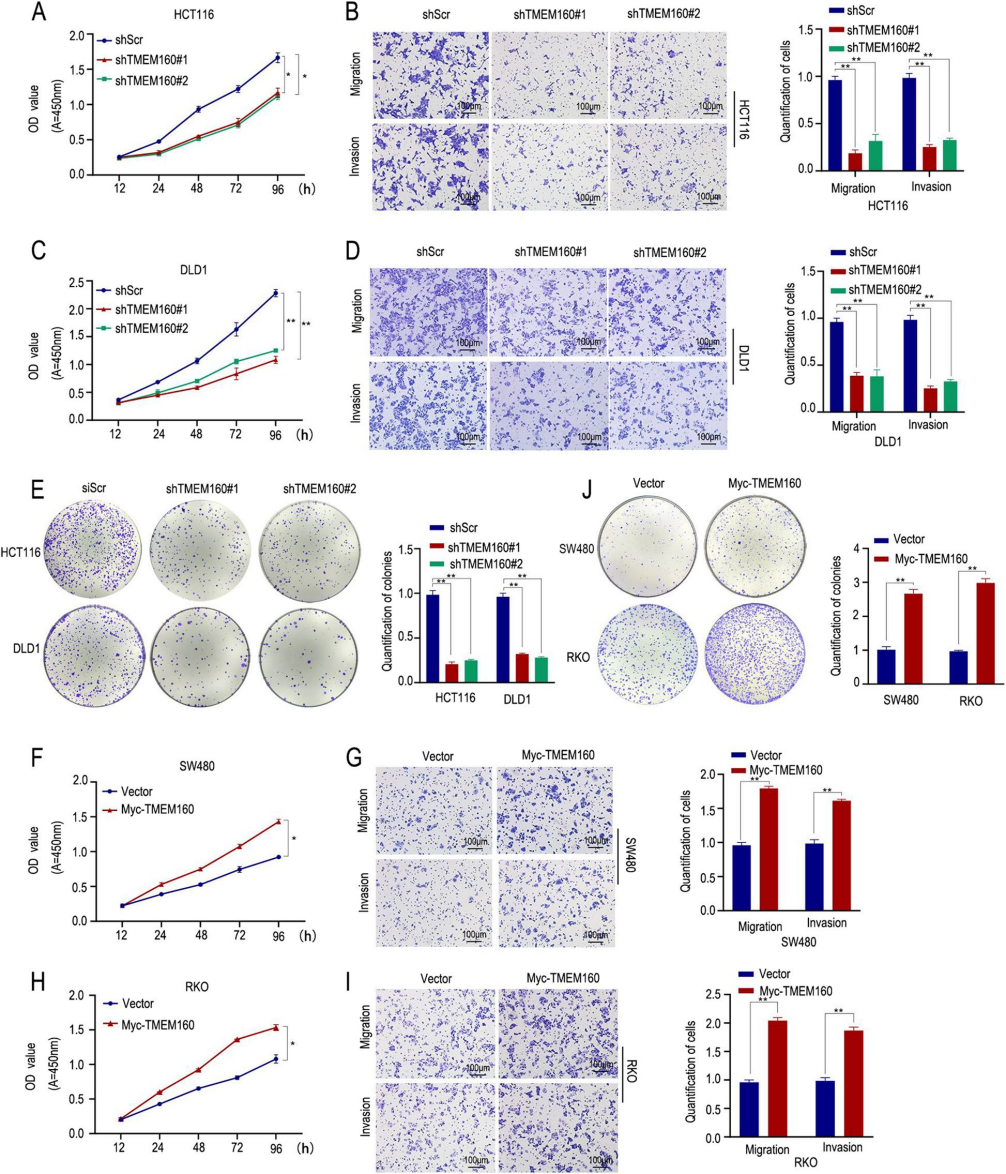
TMEM160 promotes malignant biological behavior in CRC cells. After the knockdown of TMEM160 in HCT116 and DLD1 cells using siTMEM160#1 and siTMEM160#2. **A**, **C** The CCK-8 assay demonstrated that cell proliferation was inhibited when TMEM160 was reduced. **B**, **D** The results of the transwell assay indicated a significant reduction in both cell migration and invasion. **E** The colony formation ability of CRC cells was significantly impaired when TMEM160 expression was downregulated. **F**-**J** SW480 and RKO cells that were stably transfected with Myc-TMEM160 plasmids exhibited significant enhancements in CRC cell proliferation (**F**, **H**), migration, invasion (**G**, **I**), and colony formation abilities (**J**). Independent biological experiments were repeated three times each, helping to ensure the reliability and reproducibility of the results. Statistical significance is denoted by *p < 0.05 and **p < 0.01. Scale bar, 100 μm

The results of these experiments demonstrated that knockdown of TMEM160 significantly reduced the proliferation, migration, invasion, and colony formation abilities of DLD1 and HCT116 cells (Fig. 4A–E). Conversely, exogenous overexpression of TMEM160 promoted the cell proliferation, migration, invasion, and colony forming abilities of RKO and SW480 cells (Fig. 4F–J). The downregulation of TMEM160 impaired cancer cell growth and metastatic potential, while its overexpression promoted these phenotypic characteristics. These findings collectively indicate that TMEM160 may have a significant impact on the development and progression of CRC.

### Deletion of TMEM160 impairs the role of PD-L1 in T cell-mediated tumor cytotoxicity and reduces radiotherapy resistance in CRC cells

**Fig. 5:**
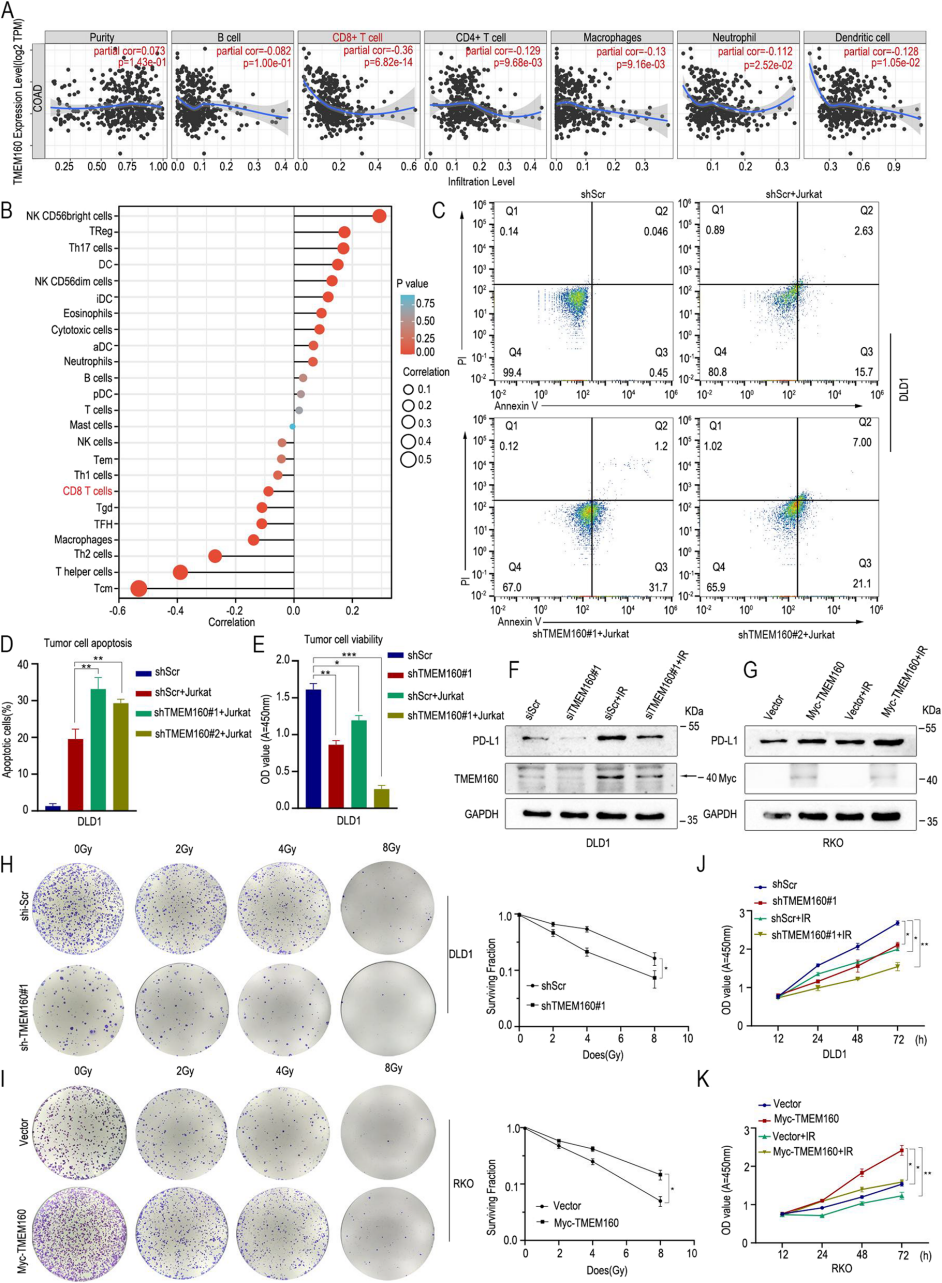
Deletion of TMEM160 impairs the role of PD-L1 in T cell-mediated tumor cytotoxicity and reduces radiotherapy resistance in CRC cells. **A** The association of TMEM160 with immunity was predicted using the TIMER database. **B** Schematic representation of TMEM160 correlation with immune infiltration. **C** FACS analysis of Jurkat cell-mediated killing of tumor cells using Annexin V and propidium iodide (PI) double staining. **D** The quantitative measurement of apoptotic cells in the Q2 and Q3 quadrants of each group, with more than 10^4^ cells counted in each group. **E** CCK8 analysis was performed to detect tumor cell viability after incubation with activated Jurkat cells. **F**, **G** To investigate the protein levels of TMEM160 and PD-L1 after radiotherapy (RT), measurements were performed in two experimental groups: siTMEM160#1 and TMEM160 overexpression. Both groups were subjected to 8 Gy of radiation. **H**, **I** The colony formation assays were visually represented through representative photographs, depicting the surviving proportions of DLD1 and RKO cells following irradiation with 0, 2, 4, and 8 Gy. **J**, **K** The proliferation of DLD1 and RKO cells was assessed using CCK8 assays. The independent biological experiments were repeated three times. Statistical significance is denoted *p < 0.05, **p < 0.01 and ***p < 0.001

Bioinformatics analysis was employed to investigate the relationship between TMEM160 and immunity. The findings revealed a significant negative correlation between TMEM160 and tumor immunity, as depicted in Fig. 5A–B. To explore the potential role of TMEM160 in regulating tumor immune suppression through its modulation of PD-L1 expression, we carried out T cell cytotoxicity assays. As depicted in Fig. 5C–E, the downregulation of TMEM160 expression resulted in increased apoptosis and heightened sensitivity to cell killing that was mediated by human Jurkat cells. These observations suggest that TMEM160 may contribute to tumor immune suppression by stabilizing PD-L1 expression.

To evaluate the biological function of TMEM160 in the radioresistance of CRC cells, we conducted experiments using DLD1 and RKO cells. In DLD1 cells, we transduced cells with either a control vector or a vector expressing shTMEM160#1 to downregulate TMEM160 expression. Conversely, in RKO cells, we used an overexpression plasmid to upregulate TMEM160 expression. RT was performed 48 h after transfection and revealed a significant increase in TMEM160 and PD-L1 levels after RT (Fig. 5F, G). The effect of TMEM160 expression on radiosensitivity was then analyzed using clone formation experiments. The DLD1-shTMEM160#1 group showed a lower survival rate after irradiation than the control group (Fig. 5H). Consistent with these findings, RKO overexpression of TMEM160 resulted in higher survival than the controls (Fig. 5I). Moreover, CCK-8 assays demonstrated that the RKO cells overexpressing TMEM160 exhibited significantly increased proliferation compared to the control group. On the other hand, the proliferation of DLD1 cells with TMEM160 knockdown was significantly lower than that of the control group (Fig. 5J, K). These data indicate that TMEM160 expression correlates with radiosensitivity and that inhibition of TMEM160 can reverse radioresistance in CRC.

In summary, the deletion of TMEM160 results in the destabilization of PD-L1 expression, leading to a reduction in the immunosuppressive function of PD-L1. Additionally, the deletion of TMEM160 enhances the radiosensitivity of CRC cells.

### TMEM160 plays a crucial role in tumor immune evasion

**Fig. 6:**
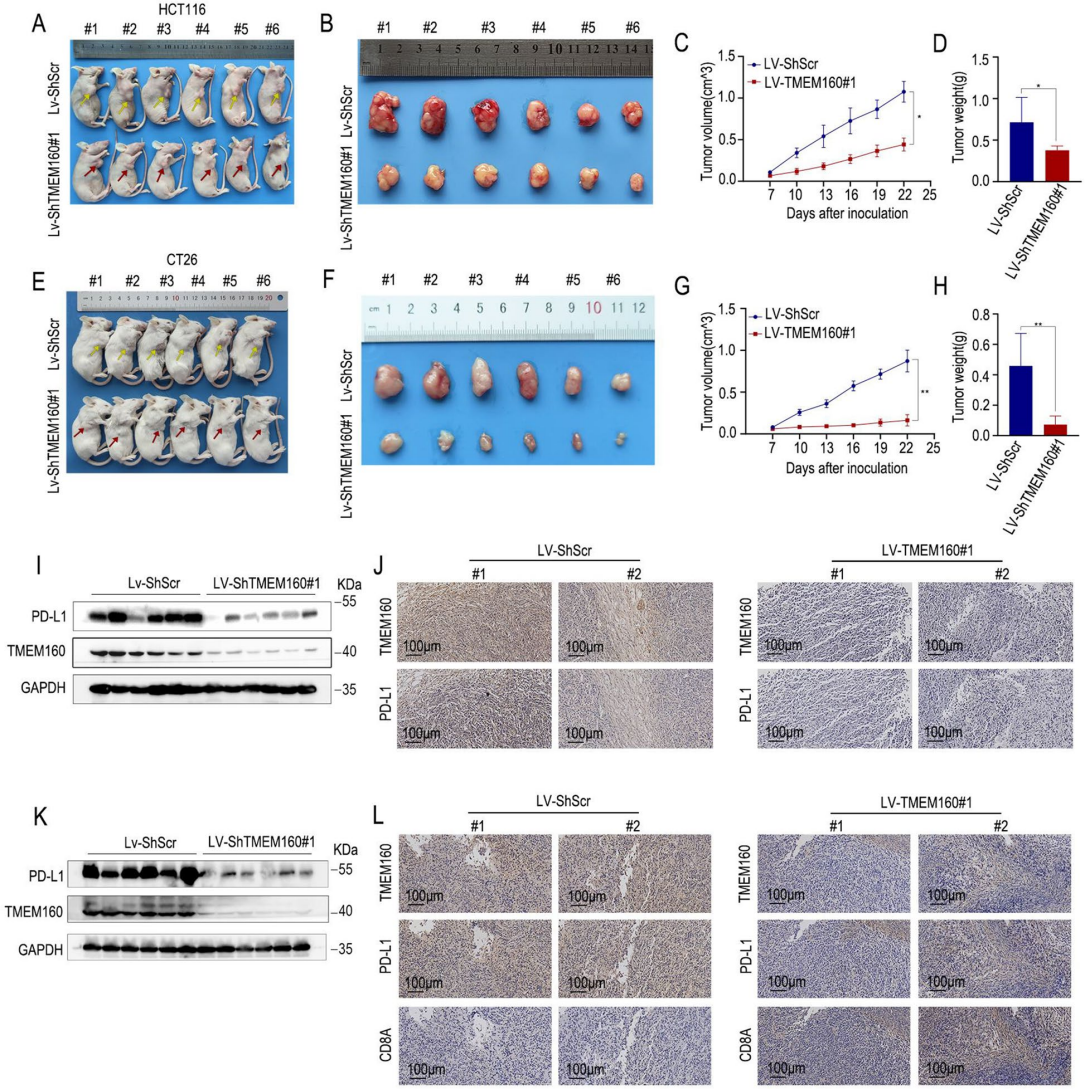
TMEM160 plays a crucial role in tumor immune evasion. **A**, **B** To establish CDX models, nude mice were subcutaneously injected with HCT116 cells that had been stably transfected with LV-ShTMEM160#1 or LV-ShScr. **C**, **D** In the LV-TMEM160#1 group, the tumor volume and weight were lower than those in the control group (mean ± SEM, *n* = 6/group). **E**, **F** To establish CDX models, BALB/c mice were subcutaneously injected with CT26 cells that had been stably transfected with LV-ShTMEM160#1 or LV-ShScr. **G**, **H** In the LV-TMEM160#1 group, the tumor volume and weight were markedly lower than those in the control group (mean ± SEM, *n* = 6/group). **I**, **J** Western blotting and IHC were conducted to detect the protein levels of TMEM160 and PD-L1 in tumor tissues obtained from subcutaneous CDX models constructed by HCT116 cells.** K**, **L** The protein levels of TMEM160 and PD-L1 and the infiltration of CD8 + T lymphocytes in subcutaneous CDX models constructed from CT26 cells were determined by western blotting and IHC

To explore the effect of TMEM160 on tumor growth, we conducted animal experiments in immunodeficient and immunocompetent mice. This study used HCT116 human and CT26 mouse CRC cells. We observed that, in severely immunodeficient NCG mice, tumor growth derived from LV-shTMEM160#1 cells was lower than that derived from LV-shNC control cells (Fig. 6A–D). Moreover, we found that LV-shTMEM160#1 significantly restricted tumor growth in immunocompetent BALB/c mice, indicating a critical role for TMEM160 in tumor immune evasion (Fig. 6E-H).

We collected subcutaneous tumor samples for western blotting and IHC to assess the expression of PD-L1 and the functionality of TILs in the tumor models. As expected, after the deletion of TMEM160, the expression of PD-L1 was significantly reduced in immunodeficient mice (Fig. 6I, J). We also observed a significant downregulation of PD-L1 expression and a significant increase in CD8 + T cell infiltration in tumors derived from LV-shTMEM160#1 cells in immunocompetent mice (Fig. 6K, L). These results suggest that the downregulation of TMEM160 leads to reduced PD-L1 expression, which activates cytotoxic T lymphocytes and promotes antitumor effects.

### TMEM160 is associated with PD-L1 expression in human CRC and predicts poor patient prognosis

**Fig. 7:**
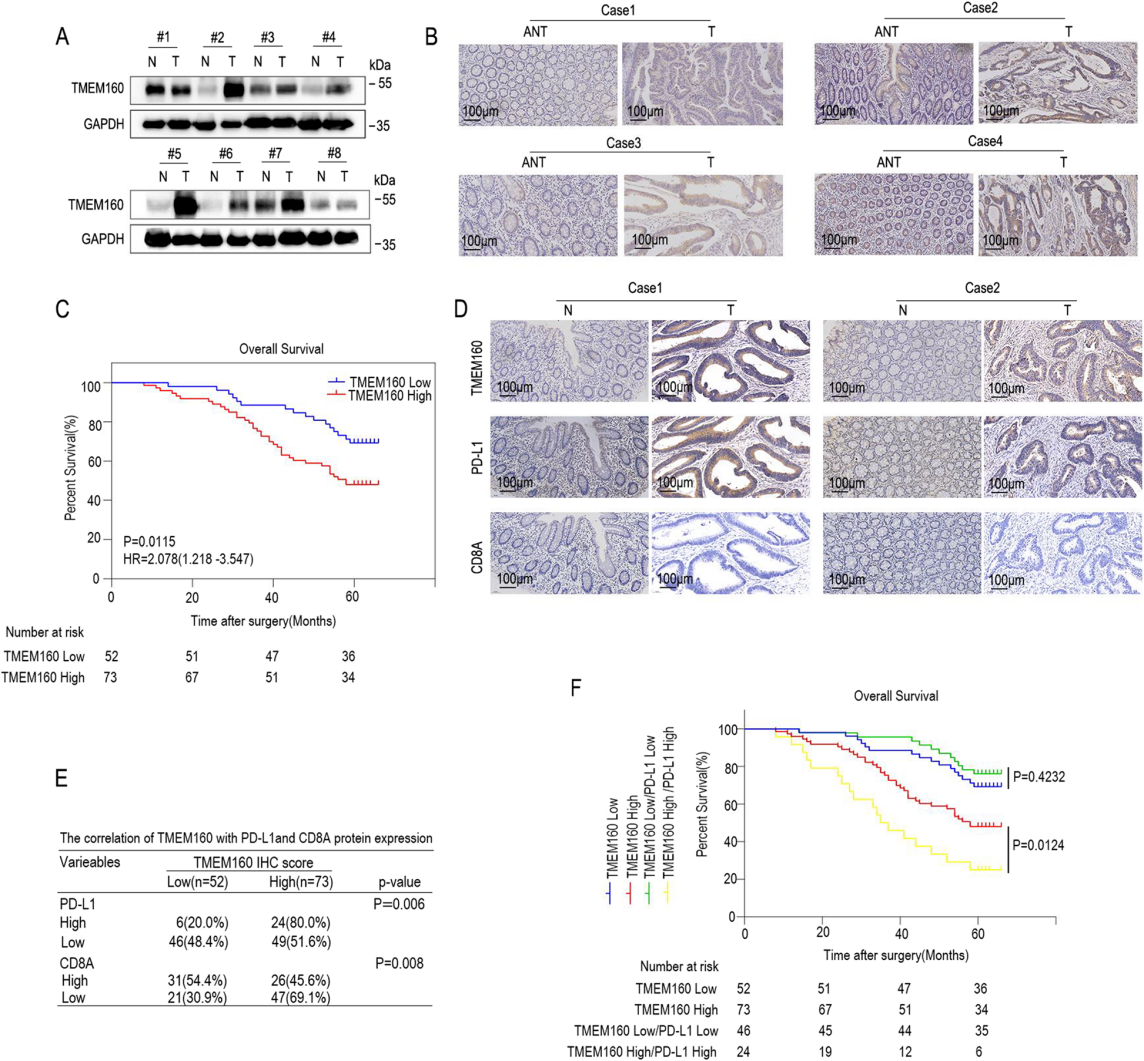
TMEM160 is associated with PD-L1 expression in human CRC and predicts poor patient prognosis. **A** Western blotting assays were employed to detect the protein levels of TMEM160 in eight pairs of CRC tissue samples. **B** IHC analysis was conducted to detect the protein levels of TMEM160 in tumor tissues (*n* = 125) and adjacent normal tissues (*n* = 125) obtained from patients clinically diagnosed with CRC. **C** The patients with CRC were categorized into groups based on the IHC results of TMEM160 protein levels. Kaplan–Meier analysis was then conducted to assess the survival outcomes in each group. **D** IHC analysis was performed on serial sections of both tumor tissues and paired normal tissues. The purpose of the analysis was to evaluate the expression levels of TMEM160, PD-L1, and CD8A proteins. To assess the correlation between TMEM160 expression and the expression of PD-L1 and CD8A, a chi-square test was employed. **E** The correlation between TMEM160 protein expression and the protein expression of PD-L1 and CD8A was analyzed using IHC assays performed on CRC tissues that were obtained from clinical patients. **F** Kaplan–Meier survival analysis was performed on a cohort of 125 patients, who were classified into four groups based on the protein levels of TMEM160 and PD-L1

To verify our findings in clinical patient samples, we performed western blotting and IHC analyses of CRC tissue and the adjacent normal tissue. The results showed higher expression of TMEM160 in CRC tissues, especially in the cell membrane and cytoplasm, than in normal tissues (Fig. 7A, B). We collected basic information from 125 patients with CRC and assessed their prognoses. The baseline characteristics are shown in Table 1. Kaplan–Meier survival analysis showed that the overall survival time of patients with high expression of TMEM160 was significantly shorter than that of patients with low or no expression of TMEM160. This finding suggested that TMEM160 holds clinical significance as a potential prognostic marker in CRC (Fig. 7C). Unadjusted Cox regression analysis showed that the depth of local invasion, regional lymph node metastasis, Tumor Node Metastasis (TNM*)* stage, and TMEM160 expression were prognostic factors for CRC (Table 2). Further multivariate regression analysis also showed that TNM stage and TMEM160 expression were independent prognostic factors (Table 2).

**Table 1:** Clinical characteristics of patients according to TMEM160

Varieables	N	Low TMEM160(%)	High TMEM160(%)	*p*-value
Gender				0.183
Female	47	16(34.0%)	31(66.0%)	
Male	78	36(46.2%)	42(53.8%)	
Age(years)				0.700
< 60	53	21(39.6%)	32(60.4%)	
≥ 60	72	31(43.1%)	41(56.9%)	
Tumor size(cm)				0.137
< 4	43	14(32.6%)	29(67.4%)	
≥ 4	82	38(46.3%)	44(53.7%)	
Differentiation status				0.914
Well + Moderate	69	29(42.0%)	40(58.0%)	
Poor and undifferentiated	56	23(41.1%)	33(58.9%)	
Depth of invasion				0.022
T1 + T2	50	27(54.0%)	23(46.0%)	
T3 + T4	75	25(33.3%)	50(67.6%)	
Lymph node metastasis				0.047
N0	59	30(50.8%)	29(49.2%)	
N1 + N2	66	22(33.3%)	44(66.7%)	
TNM stage				0.019
I +II	52	28(53.8%)	24(46.2%)	
III + IV	73	24(32.9%)	49(67.1%)

**Table 2:** Univariable and multivariable COX regression analysis of prognostic factors associated with OS

Variables	Univariable analysis		Multivariable analysis	
	HR(95CI)	*p*-value	HR(95CI)	*p*-value
Gender(female vs male)	0.833(0.473–1.468)	0.528	-	-
Age (years) (< 60 vs ≥ 60)	0.588(0.331–1.045)	0.070	-	-
Tumor size (< 4 cm vs ≥ 4 cm)	0.930(0.528–1.639)	0.803	-	-
Differentiation status (well + moderate vs poor and undifferentiated)	0.631(0.369–1.077)	0.092	-	-
Depth of invasion (T1 + T2 vs T3 + T4)	0.511(0.285–0.918)	0.025	0.981(0.530–1.819)	0. 950
Lymph node metastasis (N0 vs N1 + N2)	0.195(0.100–0.379)	< 0.0001	0.671(0.237–1.901)	0.452
TNM stage (I + II vs III + IV)	0.141(0.063–0.312)	< 0.0001	0.212(0.058–0.768)	0.018
TMEM160 (high vs low)	2.084(1.161–3.741)	0.014	1.881(1.042–3.396)	0.036

To investigate the correlation between TMEM160 and PD-L1 expression as well as their prognostic implications, we conducted IHC staining on 125 CRC tissue samples. The study findings demonstrated a positive correlation between TMEM160 and PD-L1 expression in CRC. Additionally, a negative correlation was observed between TMEM160 expression and CD8A expression. High TMEM160 expression was associated with concomitantly high PD-L1 expression and decreased CD8A expression in CRC tissues (Fig. 7D, E). Furthermore, the survival analysis conducted in this study revealed that patients with high expression of both TMEM160 and PD-L1 had shorter overall survival compared with patients with high TMEM160 expression alone (median survival time [MST] 36 vs. 58 months, *p* = 0.012) (Fig. 7F).

## Discussion

To the best of our knowledge, our study is the first to provide evidence that TMEM160 functions as an inhibitor of ubiquitination-dependent degradation of PD-L1. This novel finding holds significant implications for understanding the multifaceted role of TMEM160 in various aspects of CRC cell behavior, which includes cancer cell proliferation, migration, radioresistance, and immune evasion. Furthermore, our findings indicate a significant association between TMEM160 expression and PD-L1 expression, an association that is accompanied by a poor prognosis. Further studies may provide insights into the application of TMEM160 inhibition in immunotherapy against CRC.

The clinical response and efficacy of anti-PD-1/PD-L1 therapies have been observed to be correlated with the levels of PD-L1 expression in tumor cells [32, 33]. Therefore, it is of utmost importance to gain a comprehensive understanding of the molecular mechanisms that govern the regulation of PD-L1 expression. In this study, we first used a protein-interaction website to identify the interacting protein, TMEM160, of PD-L1 and conducted IF experiments to demonstrate that PD-L1 and TMEM160 are co-localized on the cell membrane and cytoplasm in CRC cells. Subsequently, molecular docking revealed that PD-L1 and TMEM160 can form a protein complex. To validate this interaction, we performed Co-IP and GST pull-down experiments, which provided strong evidence that PD-L1 directly interacts with TMEM160 in CRC cells. These results indicate that TMEM160 is a binding protein of PD-L1.In this study, we have not found specific sites of TMEM160 and PD-L1 binding, which requires our continued efforts.

Recently, several studies have elucidated various mechanisms involved in the regulation of PD-L1 abundance at the post-translational level. These mechanisms include degradation mediated by the proteasome or lysosomes [34, 35]. However, the precise details of how PD-L1 stabilization is regulated by the ubiquitin proteasome system remain unknown. To further elucidate the regulatory mechanisms governing the stability of PD-L1, we conducted relevant experiments.We found that TMEM160 stabilizes the protein expression of PD-L1 but does not affect its mRNA expression level. We also found that TMEM160 stabilizes the expression of PD-L1 by inhibiting its proteasomal degradation pathway.

The protein-interaction database revealed that TMEM160 can also interact with SPOP, and recent research has shown that SPOP is an E3 ubiquitination-degrading enzyme of PD-L1 [31]. First, Co-IP experiments were conducted to evaluate the interaction between TMEM160 and SPOP, which revealed an interaction between the two. Moreover, given that TMEM160 and SPOP interacted with PD-L1 and TMEM160 itself lacks a ubiquitinated domain, we hypothesized that TMEM160 and SPOP competitively interact with PD-L1. Co-IP experiments showed that the interaction between SPOP and PD-L1 was markedly reduced when TMEM160 was overexpressed. Subsequently, we expanded the ubiquitination experiment, and the results showed that TMEM160 antagonized the ubiquitination degradation of PD-L1 by SPOP, resulting in reduced PD-L1 ubiquitination. The results suggest that TMEM160 competitively binds PD-L1 with SPOP, preventing the ubiquitination degradation of PD-L1 mediated by SPOP. We speculated further that TMEM160 overexpression binds more ubiquitination sites of PD-L1, while the binding between SPOP and PD-L1 is reduced, thereby leading to a decreased ubiquitination of PD-L1.These findings enrich the regulatory mechanism of PD-L1 in CRC, and in-depth research may provide new insights into immunotherapy of CRC.

Moreover, TMEM160 is a transmembrane protein that localizes to the mitochondrial membrane [11]. It has previously been reported that loss of TMEM160 induces a UPR^mt^ and significantly increases the generation of ROS [12]; however, its effect of TMEM160 on tumor development and the immune microenvironment has not been studied. We observed that the knockdown of TMEM160 has a significant impact on the behavior of CRC cells. Specifically, when TMEM160 was knocked down, there was a significant reduction observed in cell proliferation, migration, invasion, colony formation, and radioresistance.Conversely, when TMEM160 was overexpressed in CRC cells, we observed a significant enhancement in these cellular capabilities. We also found that downregulation of TMEM160 expression increased apoptosis and enhanced sensitivity to cell death mediated by human Jurkat cells. The CRC mouse model results suggest that TMEM160 plays a critical role in tumor immune evasion and that downregulation of TMEM160 leads to reduced PD-L1 expression, which activates cytotoxic T lymphocytes and promotes antitumor effects.

CRC is indeed one of the leading causes of cancer-related deaths globally. Ataxia-telangiectasia and Rad3-related(ATR) [36], C–C motif chemokine ligand 5(CCL5) [37], and Fibroblast growth factor receptor 2(FGFR2) [38] have been shown to stabilize PD-L1 expression, thereby promoting the immune escape of CRC tumor cells. Our experiments complement these findings. Our study indicates that patients with high TMEM160 expression experience a worse prognosis compared to those with low or no TMEM160 expression. Therefore, TMEM160 may serve as a prognostic biomarker for CRC. Indeed, several studies have suggested a correlation between high PD-L1 expression and immune evasion, as well as poor prognosis [39, 40], and that high TIL infiltration within the tumor microenvironment is associated with a favorable prognosis in CRC [41]. In our study, it was observed that patients who had high expression levels of both TMEM160 and PD-L1 had a lower overall survival rate compared to those with high PD-L1 expression alone. On the other hand, patients with low expression levels of both TMEM160 and PD-L1 exhibited the most favorable prognosis. Therefore, our findings highlight the importance of evaluating the co-expression of TMEM160 and PD-L1 in predicting the prognosis of patients with CRC. In the upcoming research, we plan to collect samples from CRC patients who are either sensitive or resistant to immunotherapy and assess the expression status of TMEM160 in these patients. The aim is to investigate whether TMEM160 can serve as a biomarker for predicting the efficacy of immunotherapy in CRC patients.

## Conclusions

**Fig. 8:**
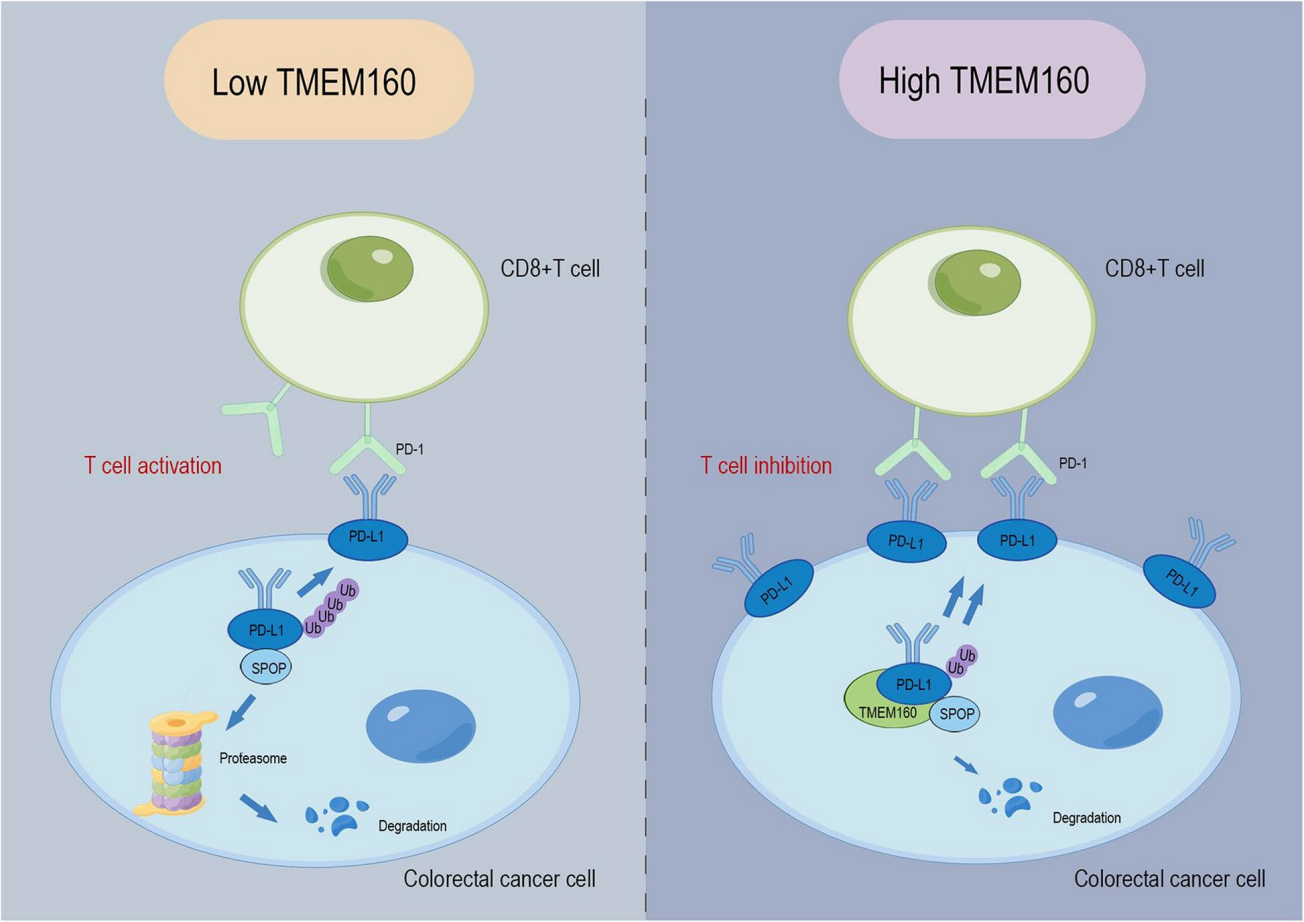
TMEM160 promotes immune escape and radiotherapy resistance to colorectal cancer cells by binding to PD-L1. In the context of CRC, the interaction between membrane-localized PD-L1 and PD-1 on T cells plays a critical role in inhibiting T cell activation. When the expression of TMEM160 is low, PD-L1 can bind to SPOP, which triggers the proteasomal degradation of PD-L1. As a result, the expression of PD-L1 localized as the cellular membrane decreased, ultimately promoting T cell activation. Conversely, when TMEM160 expression was increased in vivo, the binding of TMEM160 to PD-L1 increased, so that the binding of PD-L1 to SPOP decreased, resulting in a decrease in PD-L1 ubiquitination and stabilizing its expression, leading to immune escape and radiotherapy resistance in CRC cells. This suggests that TMEM160 expression levels can impact the stability and localization of PD-L1, ultimately influencing immune regulation and the response to radiotherapy in CRC. The figure was drawn using Figdraw

In summary, our study demonstrates that in CRC, TMEM160 reduces the ubiquitination-dependent degradation of PD-L1 by competitively binding PD-L1 with SPOP, thereby stabilizing its protein expression and promoting immune escape and radiotherapy resistance (Fig. 8). Therefore, targeting TMEM160 could be a promising strategy for enhancing the efficacy of immunotherapy in CRC.

### Supplementary Information

**Supplementary Material 1.**

## Data Availability

The authors confirm the data that has been used in this work is available on reasonable request.

## Abbreviations

TMEM: Transmembrane
PD-1: Programmed cell death protein 1
PD-L1: Programmed cell death 1 ligand 1
CRC: Colorectal cancer
SPOP: Speckle-type POZ (pox virus and zinc finger protein) protein
CD8: Cluster of differentiation 8
ICIs: Immune checkpoint inhibitors (ICIs)
UPRmt: Unfolded protein response
ROS: Reactive oxygen species
Co-IP: Co-immunoprecipitation
PBS: Phosphate Buffer Saline
CCK-8: Cell Count Kit-8
IHC: Immunohistochemistry
TPS: Tumor Proportion Score
IF: Immunofluorescence
PTMs: Post-translational modifications
TNM: Tumor Node Metastasis
MST: Median survival time
