# Supplementary material for: TMEM160 promotes tumor immune evasion and radiotherapy resistance via PD-L1 binding in colorectal cancer
Source: Cell Commun Signal. 2024 Mar 7;22:168. doi: 10.1186/s12964-024-01541-w (PMC10921666; doi:10.1186/s12964-024-01541-w)

The raw data of Fig1 is as follows:

E

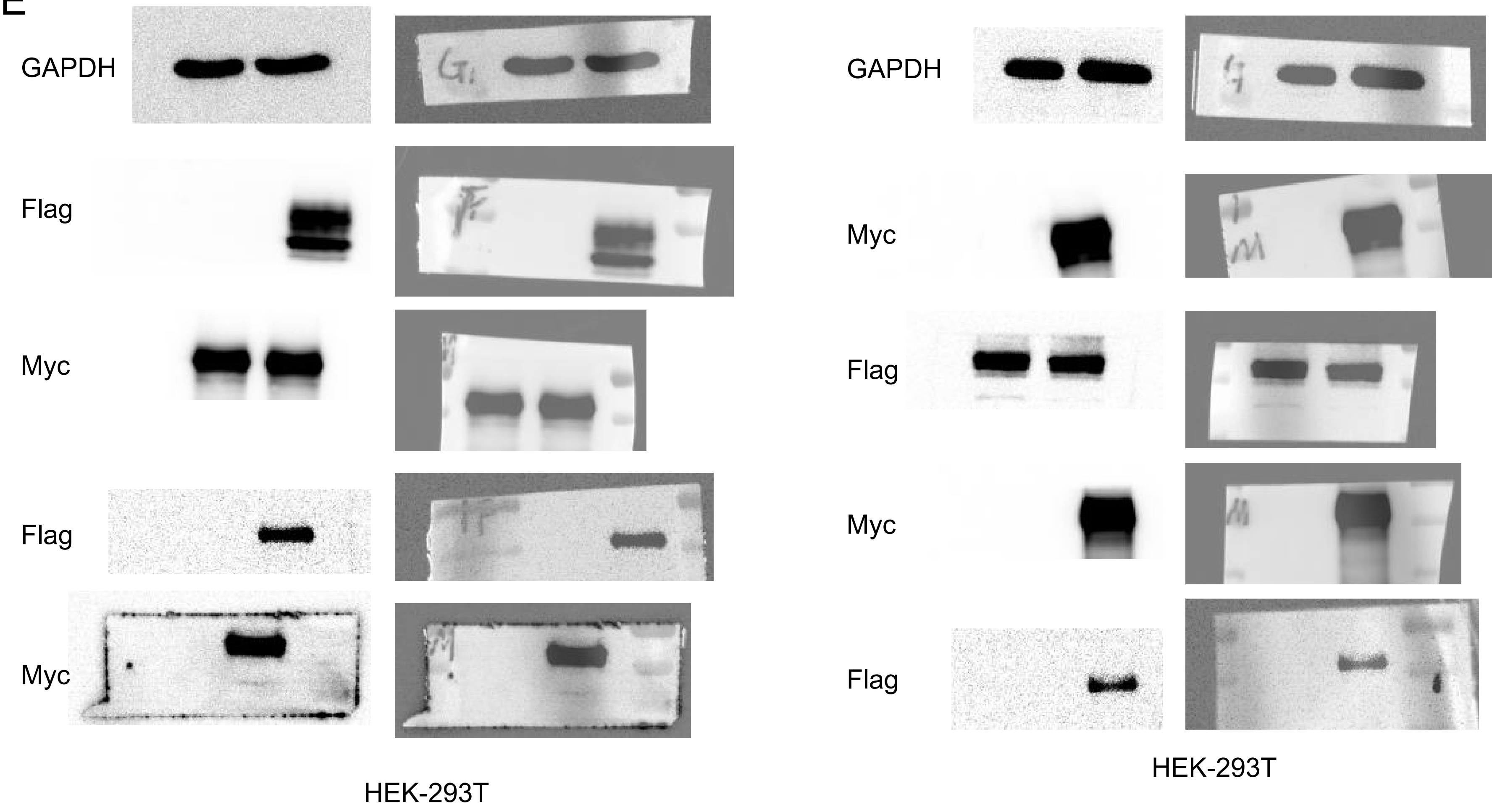

F

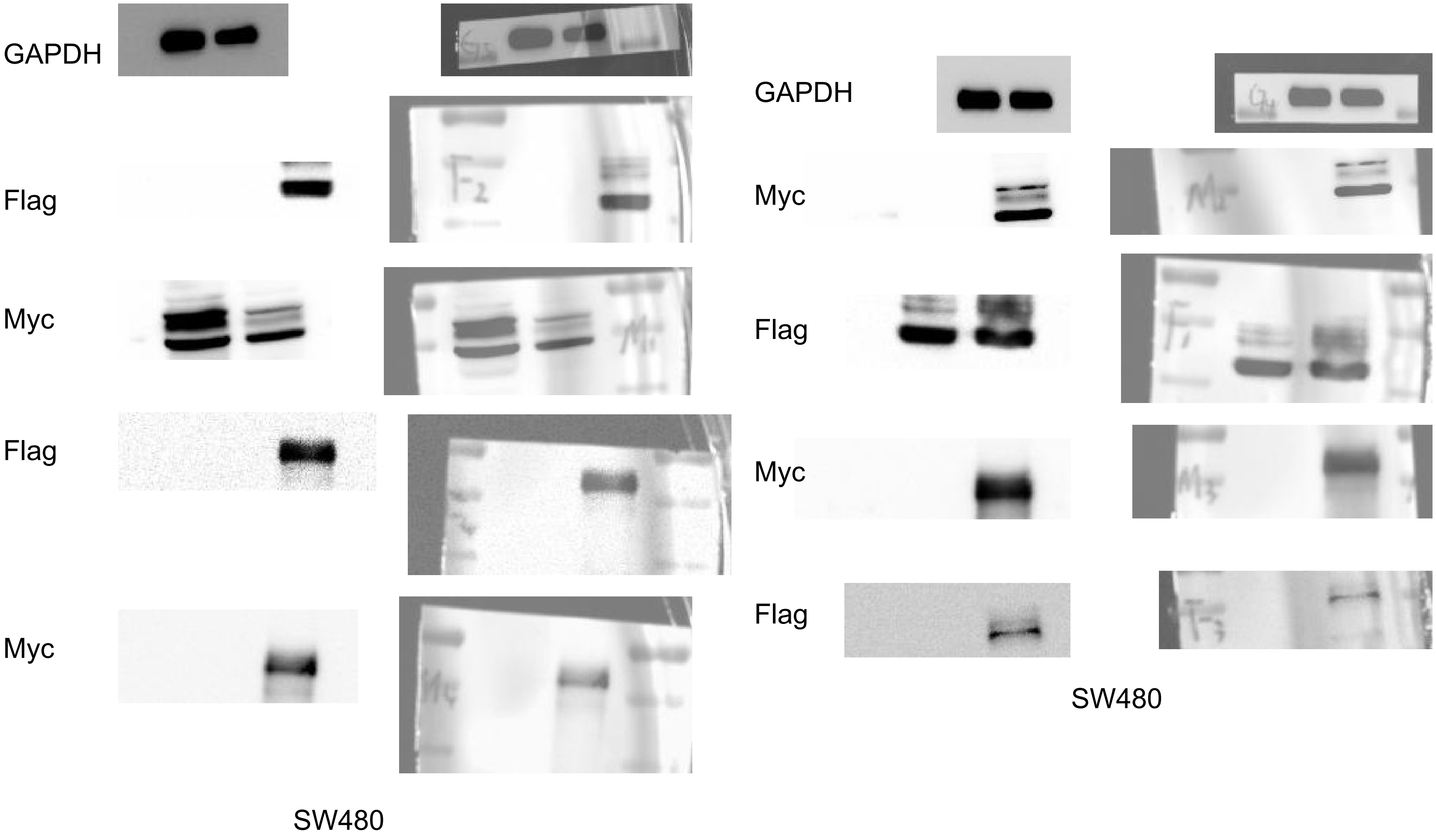

G

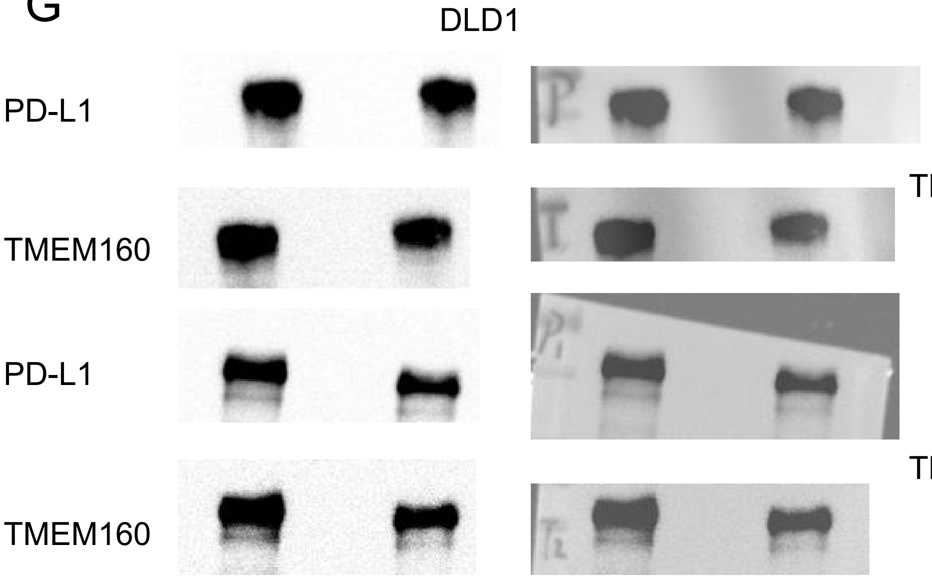
DLD1
PD-L1
TMEM160
PD-L1
TMEM160

H

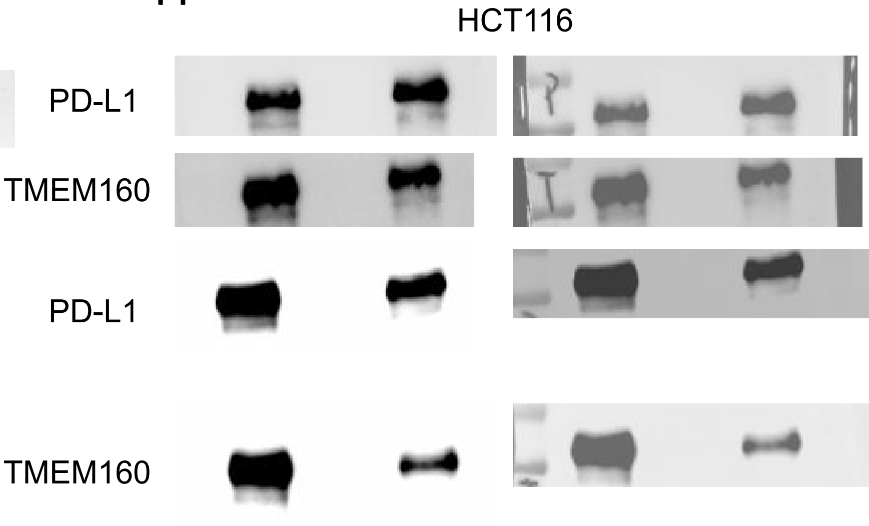
HCT116
PD-L1
TMEM160
PD-L1
TMEM160

I

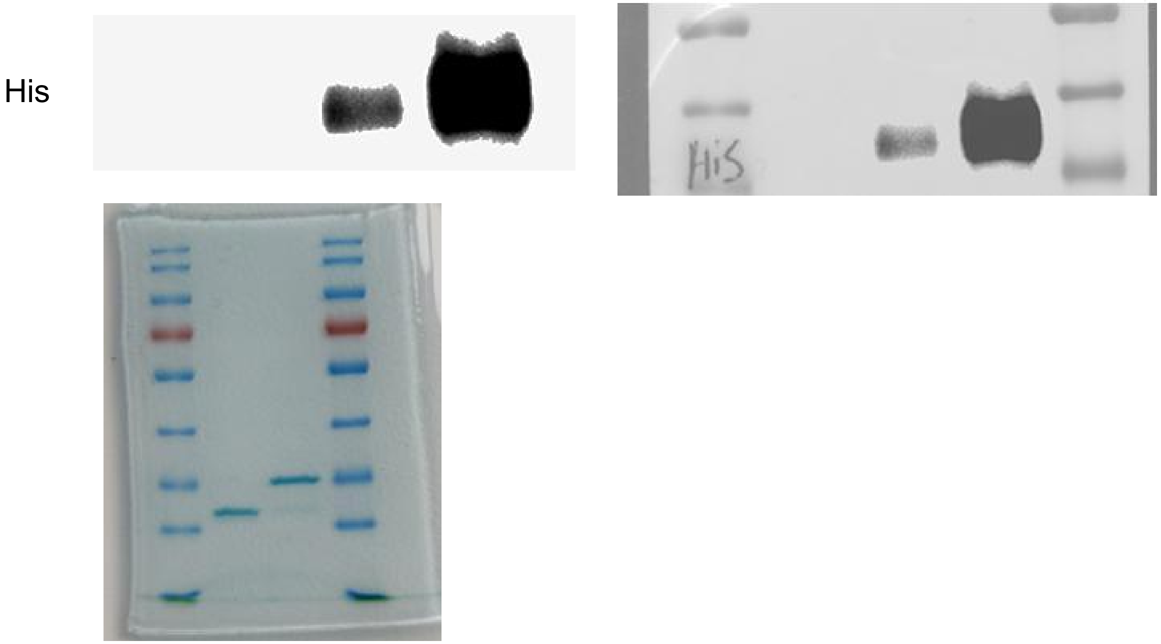
His

The raw data of Fig2 is as follows:

A

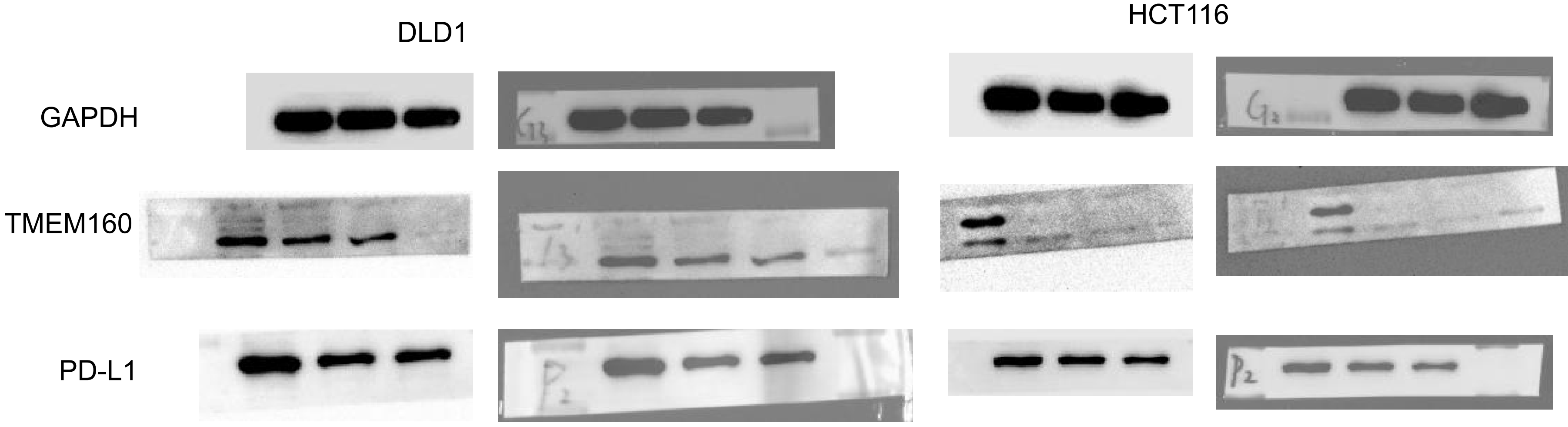


B

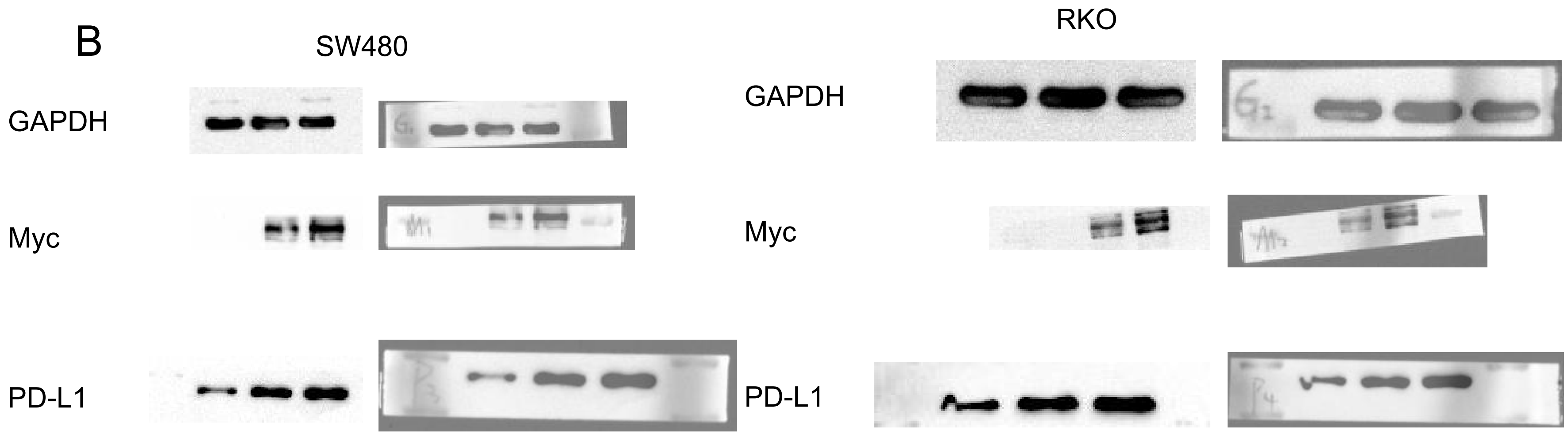

F

DLD1

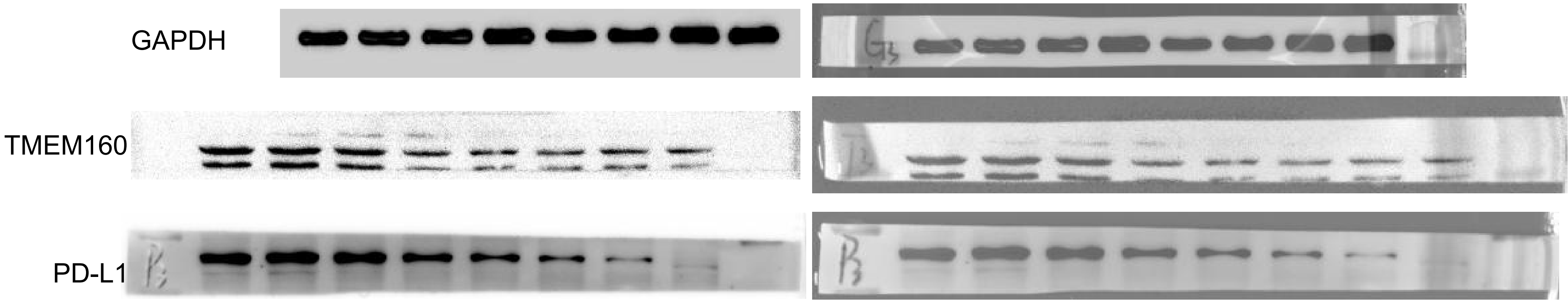


G

SW480

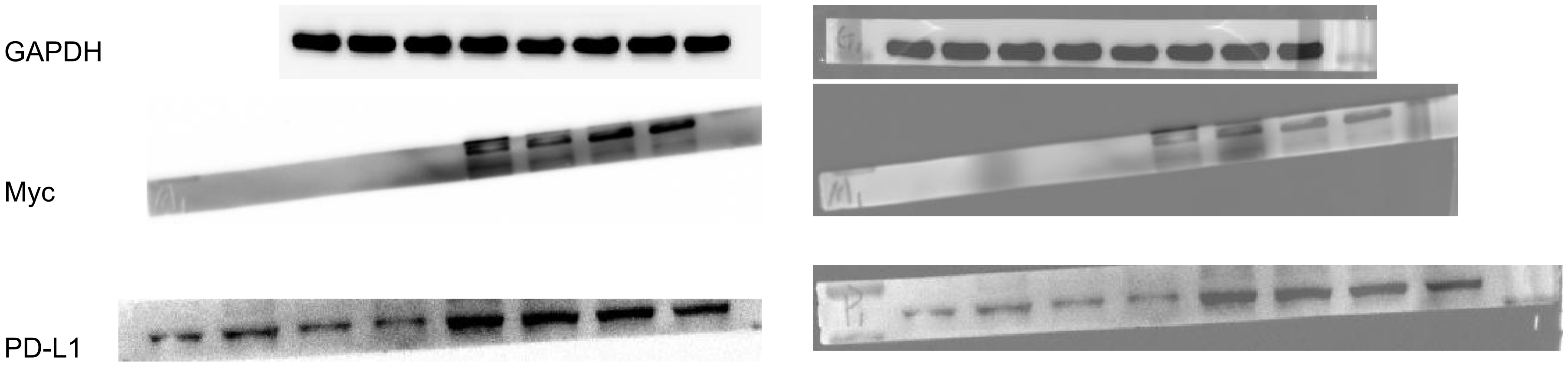

H

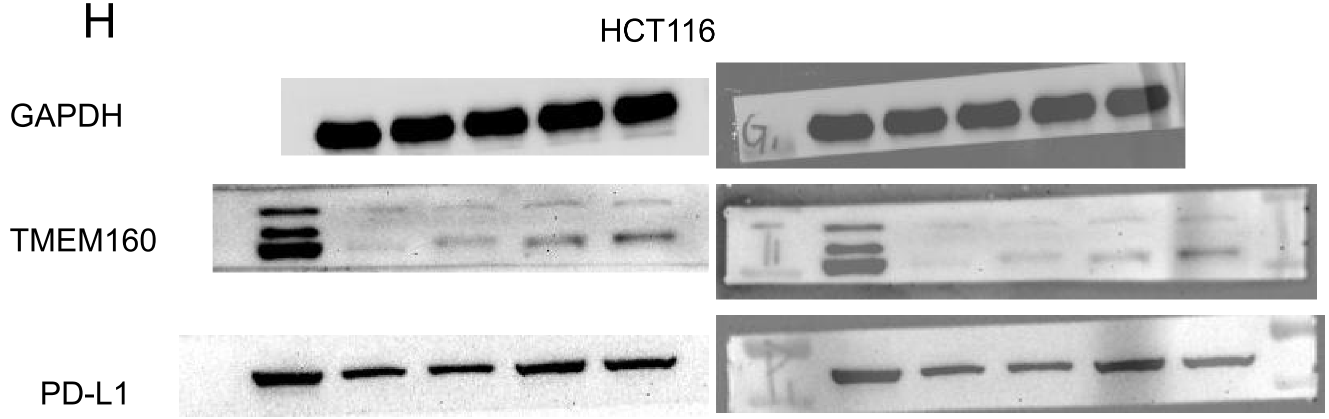


G

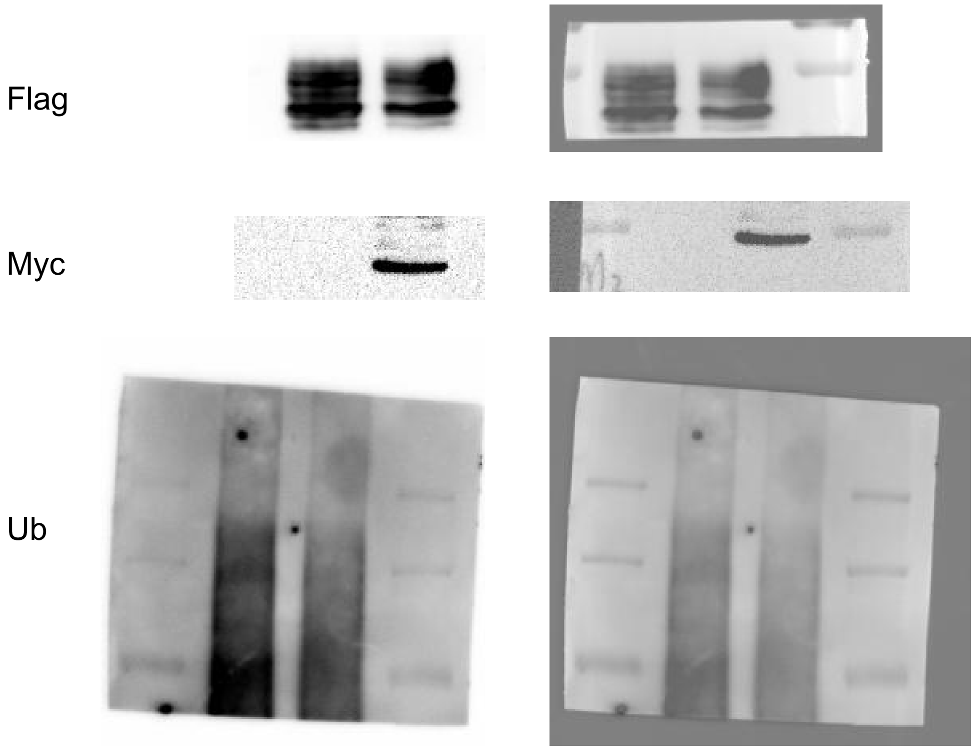

The raw data of Fig3 is as follows:

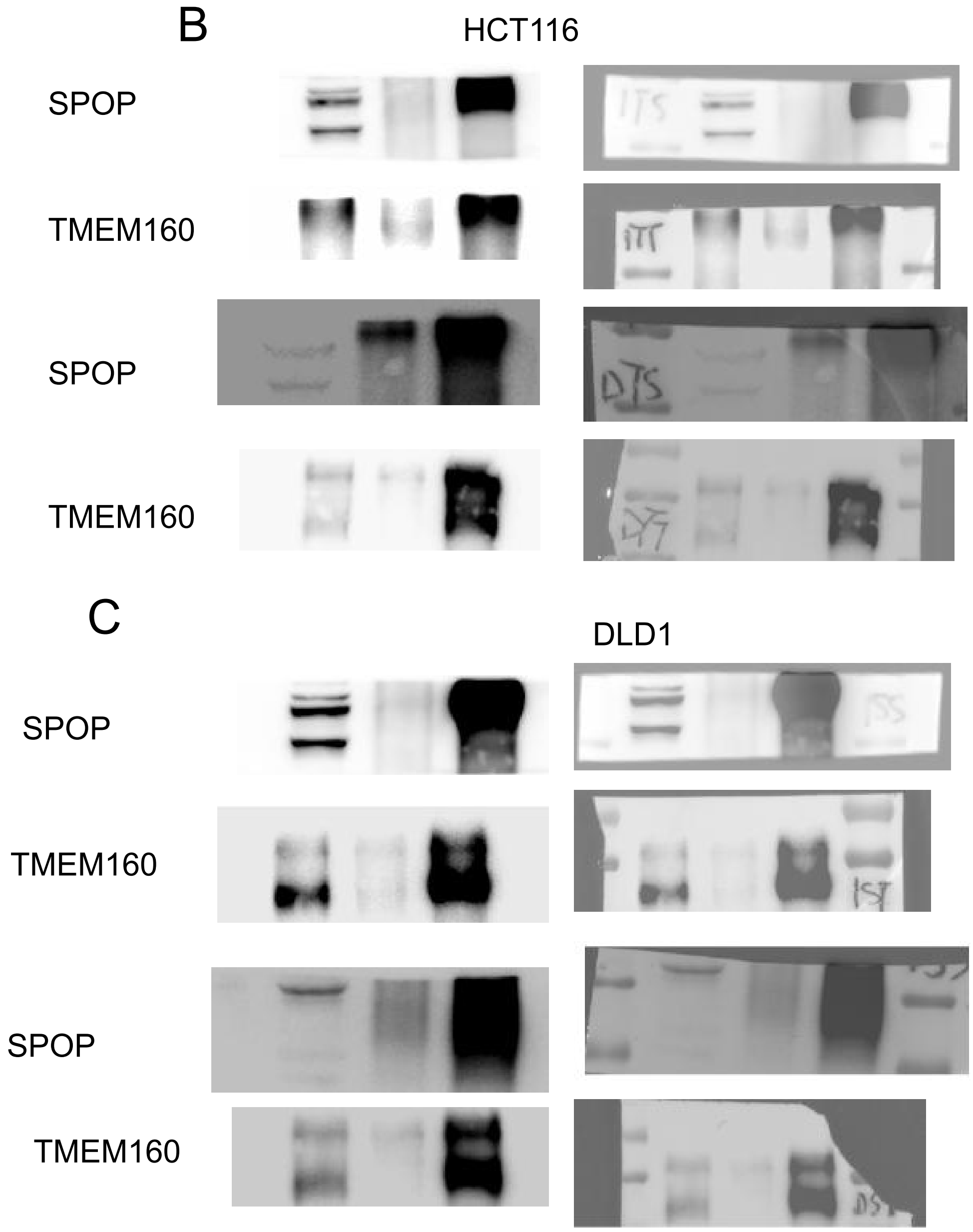

The raw data of Fig3 is as follows:

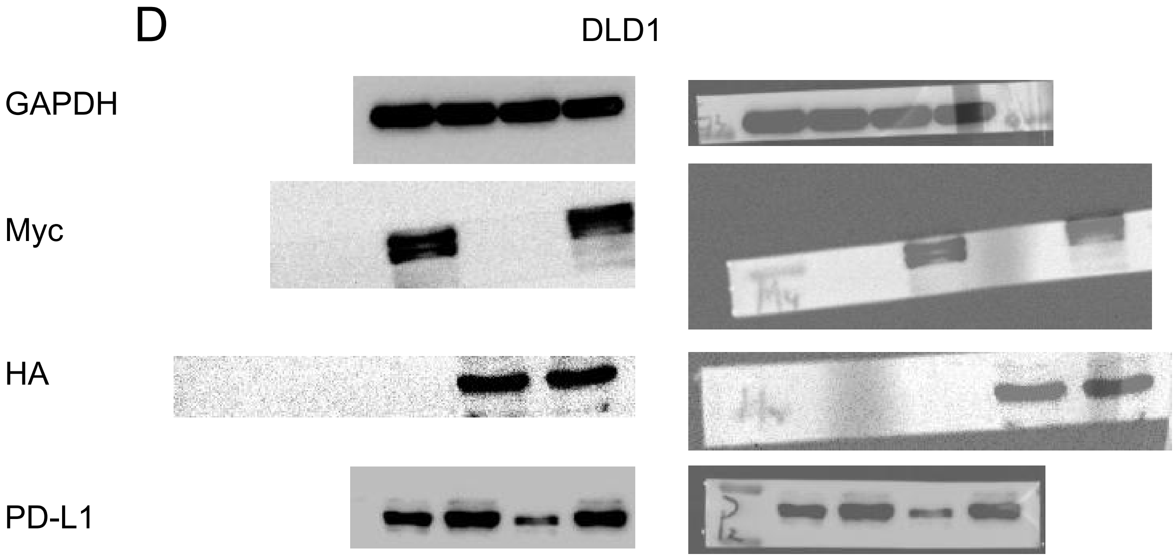


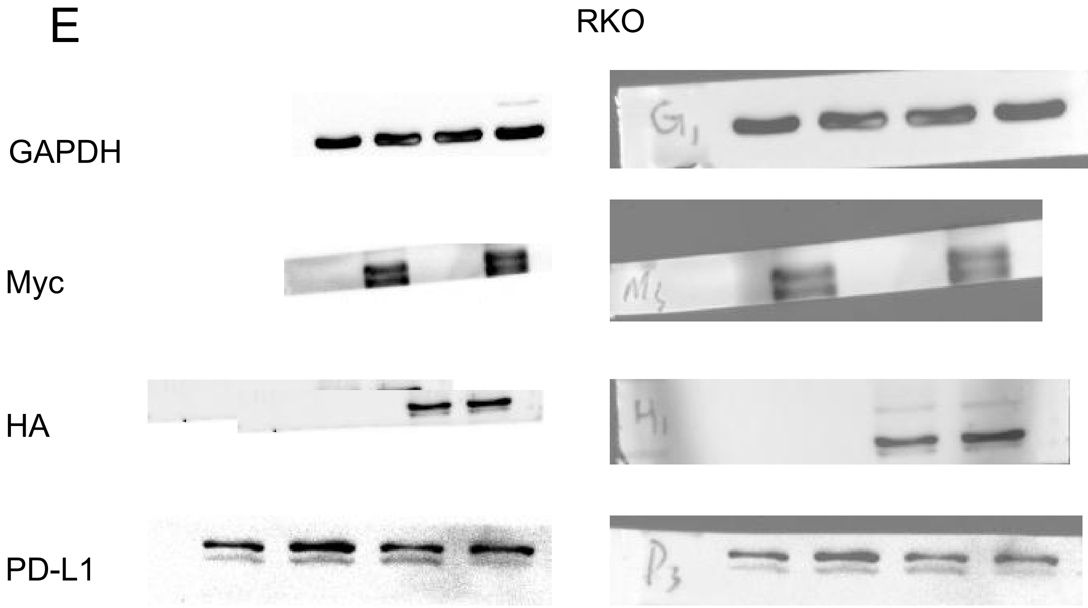

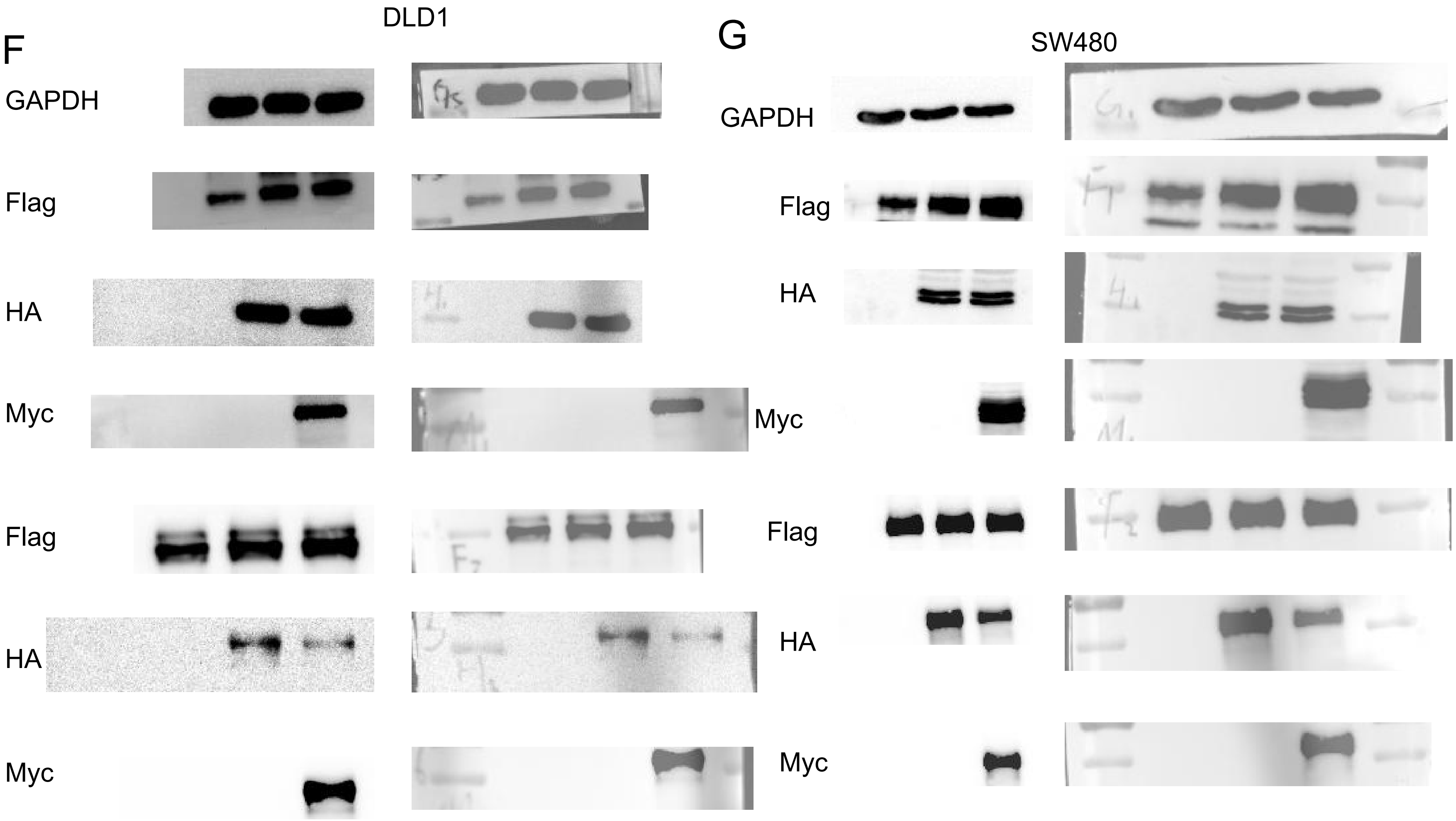
F
DLD1
GAPDH
Flag
HA
Myc
Flag
HA
Myc
G
SW480
GAPDH
Flag
HA
Myc
Flag
HA
Myc

H

HCT116

Flag

HA

Myc

Ub

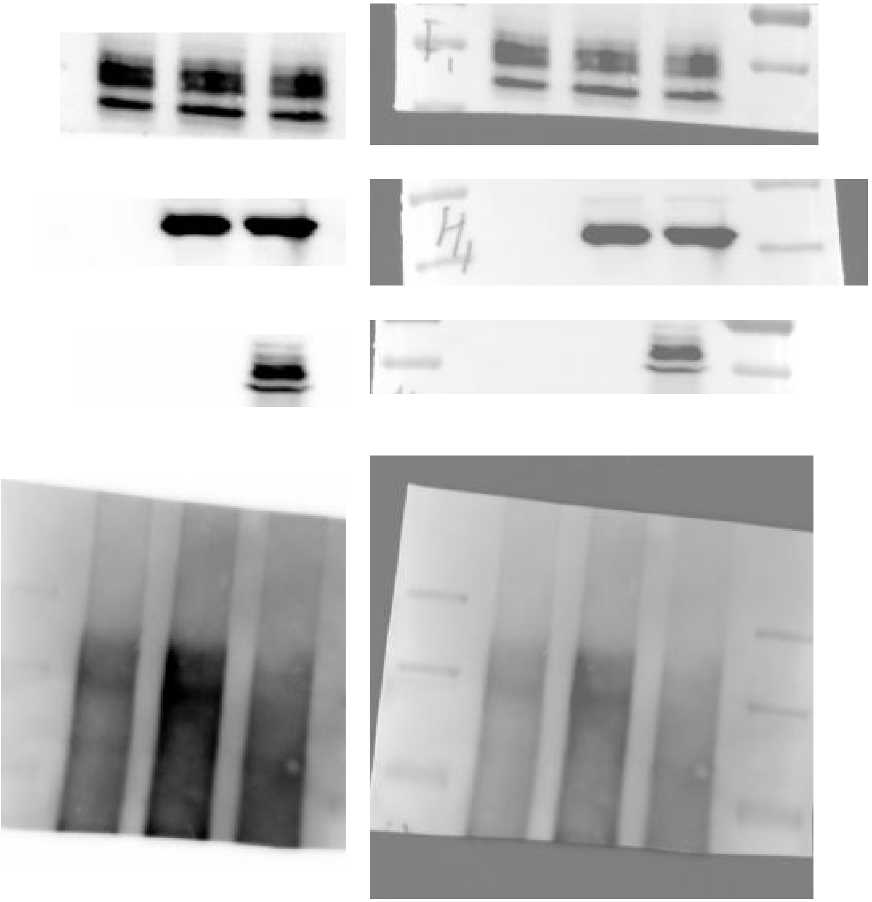

The raw data of Fig5 is as follows:

F

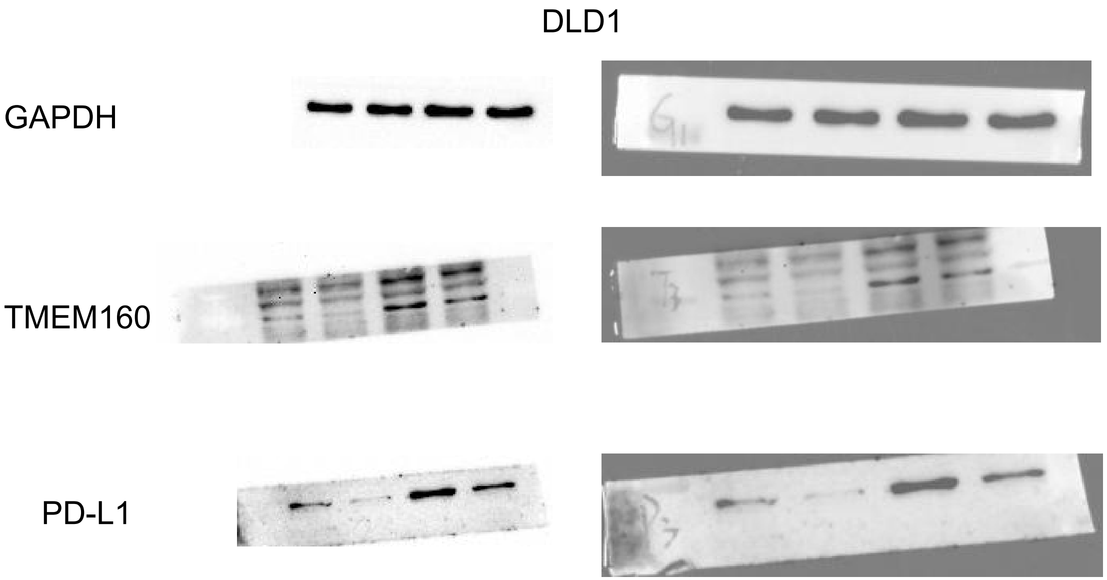


G

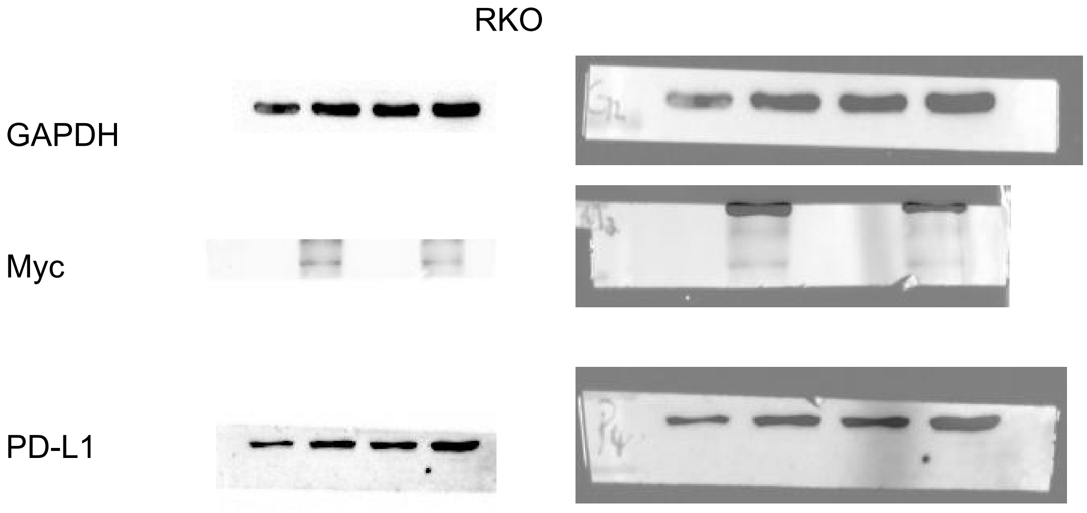

The raw data of Fig6 is as follows:

E

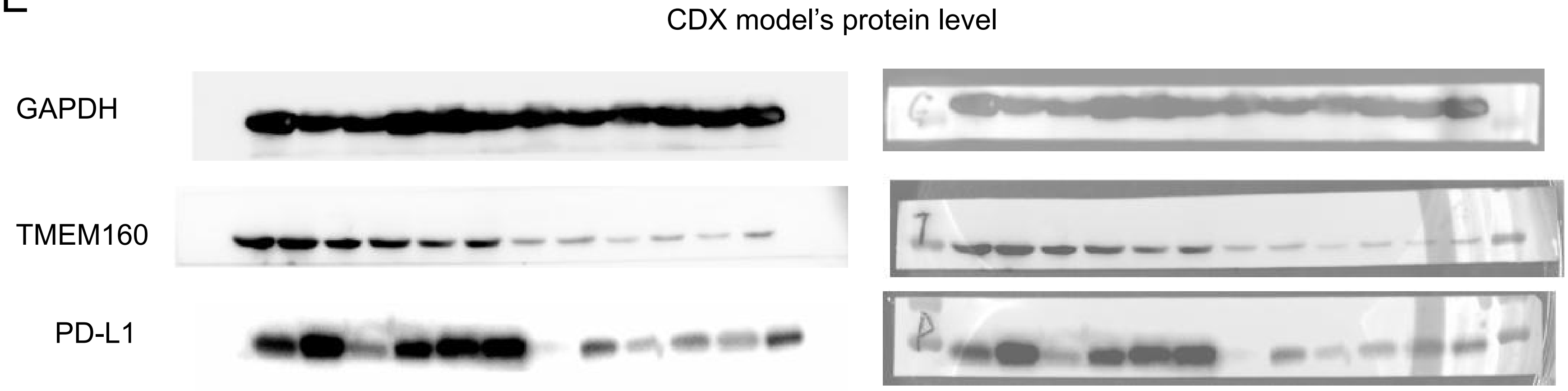


The raw data of Fig7 is as follows:

A

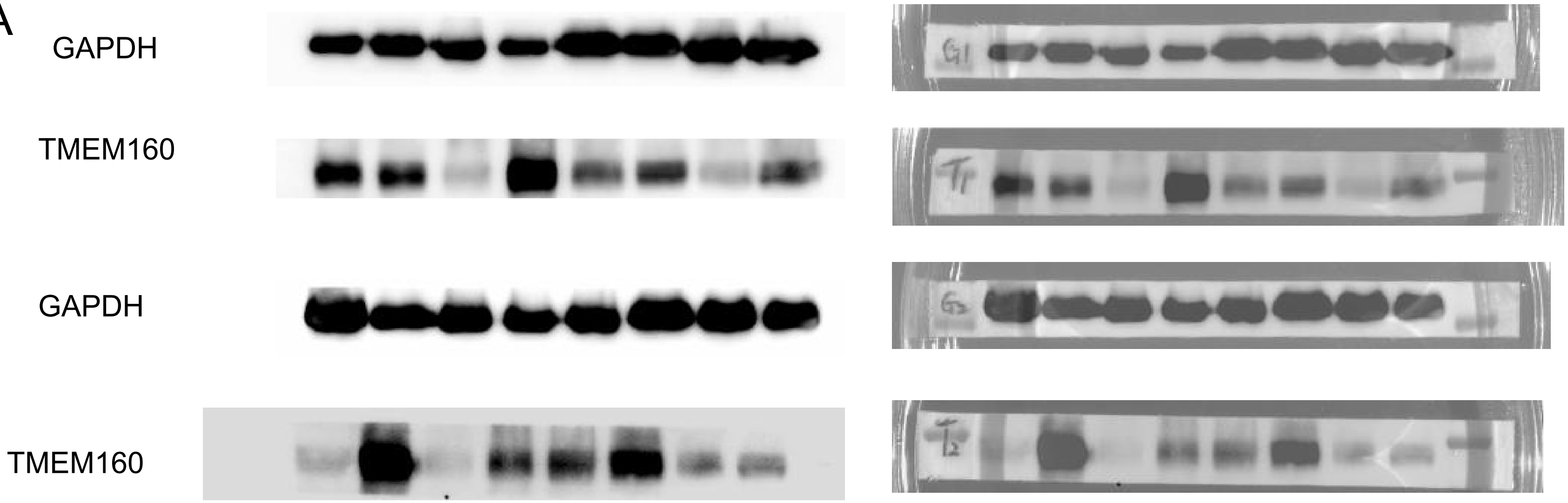

Supplement: Supplementary file 1 — Supplementary Material 1. [file 12964_2024_1541_MOESM1_ESM.pdf]
